# Topological Characteristics of the Hong Kong Stock Market: A Test-based *P*-threshold Approach to Understanding Network Complexity

**DOI:** 10.1038/srep41379

**Published:** 2017-02-01

**Authors:** Ronghua Xu, Wing-Keung Wong, Guanrong Chen, Shuo Huang

**Affiliations:** 1 City University of Hong Kong, Hong Kong; 2 Asia University, Taiwan, Lingnan University, Hong Kong; 3 City University of Hong Kong, Hong Kong; 4 Hong Kong Baptist University, Hong Kong

## Abstract

In this paper, we analyze the relationship among stock networks by focusing on the statistically reliable connectivity between financial time series, which accurately reflects the underlying pure stock structure. To do so, we firstly filter out the effect of market index on the correlations between paired stocks, and then take a *t*-test based *P*-threshold approach to lessening the complexity of the stock network based on the *P* values. We demonstrate the superiority of its performance in understanding network complexity by examining the Hong Kong stock market. By comparing with other filtering methods, we find that the *P*-threshold approach extracts purely and significantly correlated stock pairs, which reflect the well-defined hierarchical structure of the market. In analyzing the dynamic stock networks with fixed-size moving windows, our results show that three global financial crises, covered by the long-range time series, can be distinguishingly indicated from the network topological and evolutionary perspectives. In addition, we find that the assortativity coefficient can manifest the financial crises and therefore can serve as a good indicator of the financial market development.

Small-world^1^ and scale-free^2^ properties are two universal features found in analyzing real-world complex networks. With some distinguishing characteristics inheriting from regular networks and random networks, the analysis of complex networks as a powerful methodology penetrates into different disciplines, such as computer science, sociology and biology^3^. In the field of financial economics, some financial systems have also been modeled as complex networks, to better manage their complexity composed of a large number of interacting subsystems and individuals^4^. Notice that different assets present turbulent financial time series, making the market behaviors even more difficult to be examined. Therefore, an emerging direction of research at the frontiers of both economics and physics aims to provide a more fundamental understanding of the financial systems, as well as to provide practical insights for policymakers and practitioners from the network science perspective^5^,^6^,^7^,^8^.

In modeling financial systems and constructing networks from financial time series data, some “similarity” measures are needed to estimate the “connectedness” of pairs of time series. These measures could be correlation, causality, mutual information, time-delay based metrics and so on (see refs 9,10), but the most commonly used and effective one is the linear correlation^11^,^12^,^13^. Mantegna^14^ initiated a correlation-based stock network model to study the global market structure, injecting fresh air into econophysics. Subsequent research work shows that the hierarchical structure of New York Stock Exchange cannot be approximated by a random market model^15^,^16^, whereas the topologies of stock networks can be used to validate or falsify various simple but widespread market models^17^. On the other hand, the 2008 financial crisis has highlighted the main limitations of standard financial and economic models, as they cannot detect the 2008 crisis even by using posterior data. Stiglitz, winner of Nobel Prize in economics, recently points out that the standard economics models can neither forecast the crisis nor contemplate the possibility of deep downturns in financial economics^18^. Therefore, there has been urgent need of research to delve into reliable indicators of financial crisis through modeling and analyzing the complex economic networks^19^,^20^.

In order to construct a financial network and analyze its topology, a crucial step is to filter out some insignificant or even falsely associated stock pairs from the stock market^21^,^22^,^23^. This can be done by either setting a fixed threshold of correlation values or by setting a fixed number of links that represent the most closely connected stock pairs in the market data. For example, by removing the so-called relative unimportant edges, Minimal Spanning Tree (MST) method could filter out some non-arterial information of the market and focus on the most correlated ***N*** − **1** links (where ***N*** denotes the number of nodes in the network)^14^. Similar to MST, the Planar Maximally Filtered Graph (PMFG) technique is less drastic by keeping more edges of less-correlated stock pairs in order to reveal more internal market structures^21^. Besides, the correlation value threshold (or *C*-threshold for short) filtering is another popular method^11^,^23^,^24^,^25^, which could avoid the loss of important edges of high correlation values. Moreover, recent research work suggests that the selection of threshold values should fit the distribution of correlation coefficients and set the thresholds based on the mean and the standard deviation^13^.

On the other hand, the cross-correlations estimated from real data are unavoidably affected by the statistical uncertainty due to the finite size of the sample or the measurement errors^26^. Therefore, recent research also delves into selecting statistically reliable information from the correlation matrix through some statistical measures, such as the techniques based on the concepts of random matrix theory^20^,^25^ and the non-parametric surrogate data methods^10^. By applying statistical measures, the accuracy of estimating the overall network connectivity can be improved in better reflecting the underlying structure of a system. Through testing and comparing with the existing filtering methods, one motivation of this paper is to propose a new test-based approach that can filter out insignificant correlations but keep only the significantly correlated stock pairs in order to construct a reliable stock network. Moreover, this approach is designed to understand the network complexity by accounting for the distribution of the correlation coefficients and setting a unified threshold (other than the correlation value threshold) accordingly.

Apart from the static network architectures, research has also been devoting to the dynamic topologies of financial networks via a moving (rolling or sliding) window approach^13^,^14^,^22^. With emphasis on the economic implications of topological variations, a series of longitudinal stock networks are constructed covering different financial situations in the market, including bull runs, bear markets, and “business as usual” normal periods. It is shown that the changes of topological structures in the dynamic stock networks could explain the development of the economic states, especially during the periods of financial crises^21^,^22^. According to these dynamic analyses, when the market undergoes depressions, the stock networks tend to become tighter or even synchronized^13^, and the scale-free property is disrupted as compared with the ex-ante and ex-post networks^4^,^23^. In particular, a recent study reports that the stock networks could become more disassortative when the market goes through some dark periods^27^. However, all these findings are obtained from the stock networks filtered by conventional methods, either graph-based or *C*-threshold filtering methods. It is still unknown if these findings can also be validated by our newly proposed method, which is briefly mentioned above. Therefore, the other motivation of this paper is to verify the proposed approach and compare it with existing methods in terms of both static and dynamic performances.

In this paper, we focus on the statistically reliable connectivity between financial time series, which can reflect the underlying stock market structure more accurately. On the other hand, we are interested in a filtering approach that can take into account the distribution of correlation values (measuring the connectivity), and can also be used easily. As for attaining the purity of the correlated stock pairs, we adopt existing partial correlation methods, as many studies do^8^,^24^,^28^, to filter out the effect of market index on the correlations by conditioning on the market index. By removing the market effect (if it exists, measured by the market index in this paper) and obtaining the partial correlations (or conditional correlations), we show that some stocks are positively and significantly correlated mainly because of the market effect. And, when the market effect is removed, most of them become insignificant or less significant. As for attaining the econometrical reliability of an edge in the network model, we propose new *P*-threshold approaches, including both unconditional and conditional approaches, for removing the insignificant or even falsely associated stock pairs (as the filtering criteria are determined by the *P* values in the hypothesis test, this method is called *P-*threshold for short). It contributes to better understanding the network complexity by retaining the significantly correlated pairs from the stock network. We furthermore verify the proposed *P*-threshold approaches on the Hong Kong stock market by analyzing both the giant static network and the evolutionary dynamic networks.

Compared to the *C*-threshold filtering approach, the conditional *P*-threshold approach presents advantages in filtering out the statistically reliable and purely correlated stock pairs of either higher or lower correlation values; therefore, the resulting stock network can reflect the well-defined hierarchical structure of the stock market. Besides, with respect to the topological dynamics from the evolutionary market, three financial crises, covered by the long-range time period of the data, can be distinguishingly identified in terms of the average correlation, clustering coefficient, small-world property and degree assortativity. Interestingly, the dynamics of degree assortativity prove to be meaningful in revealing economic changes in the sense of positive co-movement behaviors of homogeneous stocks and negative co-movement behaviors of heterogeneous stocks. Therefore, it can serve as a good indicator of the financial market development.

## Results

To investigate the cross-correlation between any pair of individual stocks and to understand the underlying structure in the whole market, we use as many stocks as we can get from the available databases. On the other hand, we try to get the total length of the time series to be as long as possible for all stock prices being selected. With these considerations, we look for the best available datasets and obtain the daily data of 1532 stocks included in the Main Board (Excluding Depositary Receipts and Investment Companies) of the Hong Kong stock market, from January 2000 to July 2015, with 4060 trading days in total. More details about the dataset can be found in the Supplementary Information (SI) section.

In this paper, we propose the *P*-threshold filtering approaches, including both the unconditional and the conditional *P*-threshold approaches. For comparison, we also discuss the traditional *C*-threshold filtering approaches, including both the unconditional and the conditional *C*-threshold approaches. Therefore, in the first part of this section, we present the results of the giant static networks constructed by using both unconditional and conditional *P*-threshold approaches and compare them with other networks constructed by using the unconditional and conditional *C*-threshold approaches. By comparison, we will show the advantage of removing the effect of the market index and using the conditional correlation measure. We will also show the superiority of using the *P*-threshold filtering approach in better understanding network complexity. Therefore, the conditional *P*-threshold filtering approach becomes our main focus in this paper. In the second part of this section, we evaluate the effectiveness and stability of the conditional *P*-threshold approach by constructing series of evolutionary networks and presenting their dynamic mechanisms.

Before we introduce our proposed *P*-threshold approaches, we first discuss the construction of the network of financial systems by using the traditional *C*-threshold approaches. In a financial system of size ***N***, we let the set of stocks define the set of nodes ***V*** of the network, in which the number of nodes 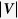 equals ***N***. There could be ***N**(**N*** − **1)/2** correlation interactions when all possible stock combinations are considered, forming the correlation matrix ***C*** with elements ***c***_***ij***_ defined in Equation (2) in the Method section. An edge in the network corresponds to an element in ***C**, e.g*., the edge ***e***_***ij***_ connecting nodes ***i*** and ***j*** corresponds to the element ***c***_***ij***_. In the unconditional *C*-threshold filtering approach, the criterion is set up by a threshold correlation value ***θ*** **(−1** ≤ ***θ*** ≤ **1)** and an edge will be chosen and added into the final network if the value of the absolute correlation is larger than the pre-determined correlation threshold; that is, if 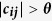.

Some studies, for example^8^,^24^,^29^, recommend using the conditional *C*-threshold approach in which the conditional correlation matrix 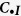, considering all possible ***N**(**N*** − **1)/2** stock combinations, is computed with elements 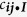 defined in Equation (6) in the Method section. Similar to the unconditional *C*-threshold approach, in the conditional *C*-threshold approach, an edge in the network corresponds to an element in the conditional correlation matrix 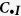 and an edge will be added into the final network if the absolute partial correlation value is larger than the pre-determined threshold; that is, if 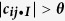.

As discussed in the Method section, the limitations of using the traditional *C*-threshold approach is that, a relatively large absolute value of the correlation coefficient could be included into the network but actually it is not statistically significant, and a relatively small value of the correlation coefficient might be discarded although actually it is statistically significant. To relax the limitation of the *C*-threshold approach, in this paper we introduce the new *P*-threshold approaches.

We first describe the unconditional *P*-threshold approach in details. We conduct the *t*-test to determine whether accepting the null hypothesis 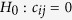 or accepting the alternative hypothesis 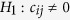, and obtain the corresponding statistics *T*_*ij*_ and *P* values *P*_*ij*_, which constitute the statistic matrices *T* and *P*. In the *P*-threshold approach, we specify a certain significance level ***α*** and add an undirected edge to connect nodes ***i*** and ***j***, if the *P* value is smaller than ***α***, and set the weight of the edge as the correlation value, as is defined in Equations (9) and (13), respectively (see the Method section). To model the stock network constructed by the unconditional *P*-threshold approach, we recommend using an undirected weighted network 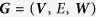, where ***V**, **E*** and ***W*** denote the sets of nodes, edges, and edge weights, respectively, and the matrix ***W*** = (***w***_***ij***_) is defined in Equation (13) in the Method section. Thus, different values of significance levels generate networks with different sets of weighted edges. Furthermore, we divide the general network ***G*** into the positive network (***PG***, describing the positive co-movement behaviors of stock pairs) and the negative network (***NG***, describing the negative co-movement behaviors of stock pairs). Both networks have the same set of nodes but “complementary” sets of edges with respect to the general network.

We now describe the conditional *P*-threshold approach in details. We recommend using the abnormal return rate of stocks and the partial correlation 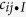 (defined in Equation (6) in the Method section) to evaluate the “pure relationship” among the paired stocks after removing the effect of the market index. We then specify a certain significance level ***α*** and perform the *t*-test to determine whether accepting the null hypothesis 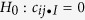 or accepting the alternative hypothesis 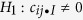, and obtain the corresponding statistics 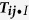 and 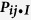 which constitute the corresponding matrices 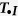 and 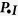. In the conditional *P*-threshold approach, we add an undirected edge to connect nodes ***i*** and ***j***, if the *P* value is smaller than ***α***, and set the weight of the edge as the conditional correlation value, as defined in Equations (12) and (14), respectively (see the Methods section). To model the stock network constructed by the conditional *P*-threshold approach, we recommend using an undirected weighted network 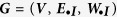, where ***V***, 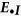 and 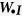 denote the sets of nodes, edges, and edge weights, respectively, and the matrix 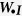 = (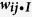) is defined in Equation (14). Furthermore, we divide the general network into the positive network (***PG***, describing the positive co-movement behaviors of stock pairs) and the negative network (***NG***, describing the negative co-movement behaviors of stock pairs). Both networks have the same set of nodes but “complementary” sets of edges with respect to the general network.

### Results of the giant static network

**Figure 1:**
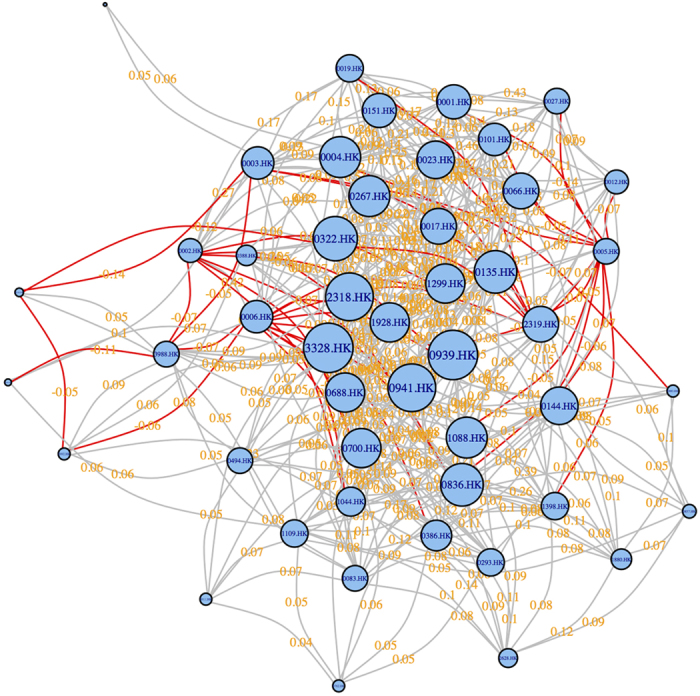
Network visualization of 48 components of Hang Sang Index (HSI) from the Main Board. The size of a node is proportional to its degree (the number of correlated stocks), where the red line denotes the negative correlation coefficients, the gray line denotes the positive correlation coefficients, and the label on the edge stands for the partial correlation coefficient that are significant at the level of 1% (with *P* values < 0.01).

The total observation period ***T*** in our paper is a long period of 15 years. Due to requirement of the assumption of zero mean and constant variance in the Capital Asset Pricing Model (CAPM) defined in Equation (3) in the Method section, we separate the long-time observed period into three equal-length shorter sub-periods, each with length of 5 years (from 2000 to 2005 as Window 1, from 2005 to 2010 as Window 2, and from 2010 to 2015 as Window 3). By comparing the constructed networks based on these three windows, we obtain consistent findings and draw the same conclusion for each window. Thus, we only present the results of Window 3 and skip reporting the results on the other two periods (Window 1 and Window 2, which can be found in the SI section). Due to the complexity of this giant network of 1,279 stocks trading on the Main Board of the Hong Kong market, we only extract a small part (containing 48 components of Hang Sang Index (HSI) from the Main Board) and exhibit it in Fig. 1 for visualization. As some of HSI components do not have sufficient co-trading days (less than two thirds of the corresponding window length mentioned in the Methods section), only 48 components remained, and the detailed symbols and names of the 48 components can be found in Table S5 in the SI section. We note that, differing from the networks constructed based on MST or PMFG, the presented network is much denser and includes a wider range of correlation values.

**Figure 2:**
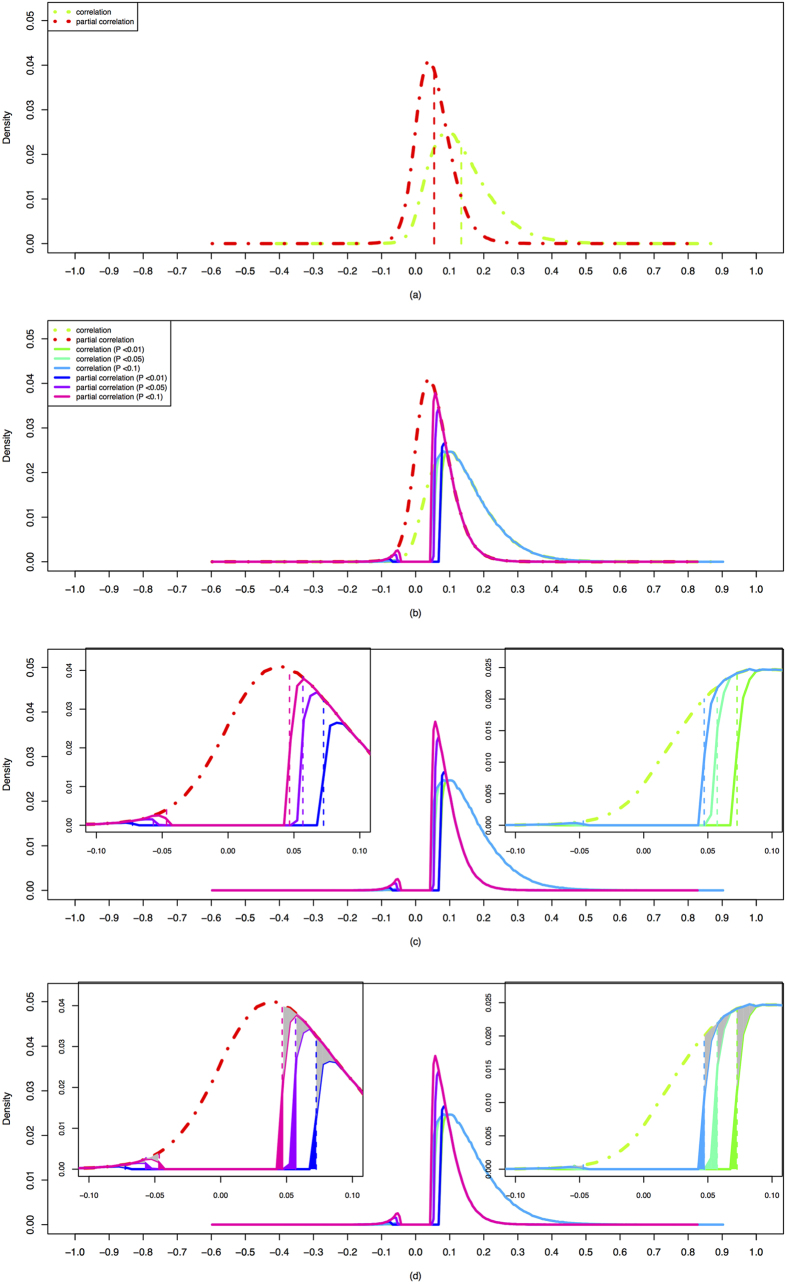
(**a**) Probability density distributions of the two types of correlation values in the giant static network of 1,279 stocks spanning from May 18, 2010 to July 23, 2015. Here, “correlation” stands for the raw Pearson correlation coefficient (defined in Equation (2)), “partial correlation” stands for the partial correlation coefficient conditioning on HSI (defined in Equation (6)). The two vertical dotted lines, the red one and the green one, denote the mean values of “correlation” and “partial correlation”, respectively. (**b**) Probability density distributions of the two types of correlation values in the giant static network of 1,279 stocks and the distributions of the filtered correlation values by the conditional and unconditional *P*-threshold approaches at three significant levels. Here, “P < 0.01”, “P < 0.05”, “P < 0.1” stand for the significance levels of 1%, 5% and 10%, respectively. (**c**) Similar to (**b**) and using the same legend as (**b**), the distributions of the filtered correlation values by the conditional and unconditional *P*-threshold approaches at three significant levels are shown in the center figure. To present a clearer view, we separate and highlight the “partial correlation” (under three significance levels) coefficients and the “correlation” (under three significance levels) coefficients in the range of [−0.1, 0.1] in the left and right insets, respectively. The vertical lines in the insets denote the correlation thresholds in the *C*-threshold approach so that the *P*-threshold approach and the *C*-threshold approach keep the same number of correlation values. (**d**) Similar to (**c**) and using the same legend as (**c**), the only difference is that, in the two insets we highlight the differences between the *C*-threshold approaches and the *P*-threshold approaches by coloring the non-intersecting regions. The regions, painted with the same color as the distributions lines, denote the smaller but significant correlation values that are excluded by the *C*-threshold approach but are included by the *P*-threshold approach. On the other hand, the regions, painted with the gray color, denote the regions of relative larger but non-significant correlation values that are included by the *C*-threshold approach but are excluded by the *P*-threshold approach.

**Figure 3:**
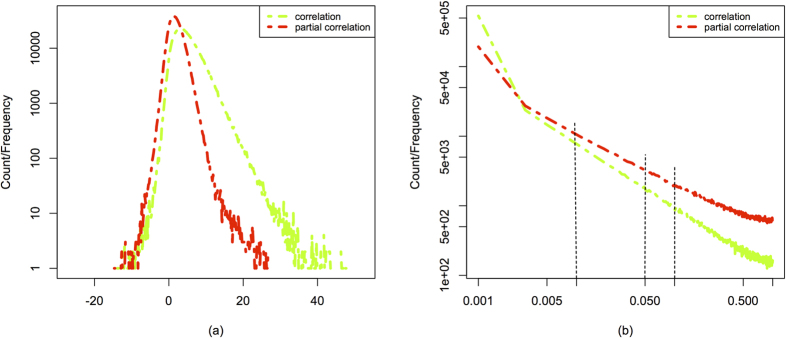
Probability density distributions of: (**a**) *t-*statistics defined in Equation (8) and Equation (11) (*y*-axis is in log scale) and (**b**) the corresponding *P* values (in log-log scale). The three vertical lines in (**b**) stand for the three *P* values or significance levels of 1%, 5% and 10% (from left to right), respectively, The red points denote the type of “correlation” and the green points denote the type of “partial correlation”.

To obtain purely correlated stock pairs, we need to examine the effect of removing the market index. For this purpose, two different correlations are compared: the unconditional correlation without removing the market effect (defined in Equation (2) in the Method section and denoted as “correlation” in Fig. 2), and the conditional partial correlation conditioning on the market index (defined in Equation (6) in the Method section and denoted as “partial correlation” in Fig. 2). We obtain all possible ***N**(**N*** − **1)/2** correlation values for any pair of stocks ***i*** and ***j***. We plot all these values in Fig. 2(a) to form the distributions of “correlation” (the dotted green line) and the “partial correlation” (the dotted red line). In the hypothesis test, the significance of the correlation coefficient is measured by both *t*-statistic and the corresponding *P* value. Similarly, we obtain all possible ***N**(**N*** − **1)/2** *t-*statistics and *P* values for all correlated stock pairs and plot these values in Fig. 3 to form the count of the “correlation” *t*-statistics and *P* values (the dotted green lines in (a) and (b)) and the count of the “partial correlation” *t-*statistics and *P* values (the dotted red lines in (a) and (b)). We note that the integral of each distribution (the area under the entire distribution) is 1 and all possible correlated stock pairs form the complete network with density of 1.

Comparing the density functions of the correlation (the dotted green line) to partial correlation (the dotted red line) in Fig. 2(a), we have the following observations: (*i*) the entire density of the correlation shifts to the left to become partial correlation; (*ii*) there are less number of positive partial correlations but more negative partial correlation coefficients (many of them could be insignificant as will be explained below) than that for the correlations; (*iii*) the mean of the partial correlation coefficients (the red vertical dotted line) is smaller than that of the correlation coefficients (the green vertical dotted line).

Besides, in order to understand the effect of significance levels on filtering statistically reliable edges, we also compare the values of the filtered correlations by applying the conditional and the unconditional *P*-threshold approaches at three different significance levels, ***α*** = 0.1, 0.05 and 0.01, respectively. We indicate these three critical values by the three vertical lines in Fig. 3(b) with *P* values of 0.1, 0.05 and 0.01, respectively. The elements on the left sides of the vertical lines represent the filtered correlation values whose *P* values are smaller than the preset significant level ***α***. We obtain all these filtered correlation values and let the number of these filtered correlated stock pairs be 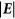 or 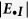. Then we plot the distributions of these filtered correlation values in Fig. 2(b), as compared to the distributions of all possible ***N**(**N*** − **1)/2** correlation values without filtering. As the integral of the latter distributions is 1, we rescale the former distributions and set the integral of the former distributions to be the proportion of 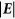 or 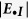 over ***N**(**N*** − **1)/2**, or equivalently, we set the integral of the distribution of the filtered correlated stock pairs to be the density of the filtered network.

Comparing the correlations and partial correlations filtered at three significance levels (refer to Fig. 2(b) and the center figure in Fig. 2(c)), we observe: (*iv*) there are more significant positive correlation coefficients than the partial correlations; *v*) when the significance level reduces from 10% (or 5%) to 5% (or 1%), the number of survived edges becomes smaller. These observations imply that some stock pairs are positively and significantly correlated due to the existence of market effect (measured by the market index in this paper) in the correlation. When the market effect is removed, most of them become insignificant or less significant.

Comparing with the conditional *C*-threshold (refer to the left insets in Fig. 2(c) and (d)) and the unconditional *C*-threshold approach (refer to the right insets in Fig. 2(c) and (d)), we further observe: (*vi*) at each significance level (10%, 5%, 1%), the filtered distributions of both correlations and partial correlations by the *P*-threshold approach are not vertical lines (which is the case in the *C*-threshold approach) but they are downward or upward sloping curves with tangent less than 90 degree. This implies: a) at each significance level, there are some relatively bigger values of correlation or partial correlation but they are not significant; b) there are some relatively smaller values of correlation or partial correlation but they are significant (refer to the colored regions in the two insets in Fig. 2(d)). In other words, these observations verify: (*I*) a relatively bigger correlation coefficient could probably be included into the network following the *C*-threshold approach, either conditional or unconditional, but actually it is not statistically significant; (*II*) a relative smaller correlation coefficient could probably be discarded from the network, but actually it is statistically significant. And we also observe: (*vii*) these two situations would happen with a higher probability when the significance level becomes smaller.

We would like to point out that our proposed *P*-threshold approach, either conditional or unconditional, retain all statistically reliable correlation coefficients, and remove all correlation coefficients that are not significant. Thereby, the filtered stock pairs by using our proposed approach will cover a wider range of truly significant correlation values. This is very different from the conventional *C*-threshold approach, either conditional or unconditional, which only cares about the stock pairs with high correlation (but could be insignificant) values.

To elaborate further about the unique characteristics of the *P*-threshold filtering approach and to support the intuitive observations with more evidence, we enlarge the ranges of the significance levels and set four different criteria with *P* < 10^−7^, <10^−5^, <10^−3^ and <10^−1^, respectively, and then compare them with the *C*-threshold approach in terms of both statistical properties and network properties. We exhibit the results of statistical properties in Table 1 and the results of network properties in Table 2. In total, there are four different types of networks filtered by the four different approaches, the type of *P*-threshold correlation filtered by the unconditional *P*-threshold approach, the *P*-threshold partial correlation by the conditional *P*-threshold approach, the *C*-threshold correlation by the unconditional *C*-threshold approach and the *C*-threshold partial correlation by the conditional *C*-threshold approach. As the conditional *P*-threshold approach is our main focus in this paper, we take the network of the *P*-threshold partial correlation as the basis (refer to Row Five to Row Eight, highlighted by the bold-faced numbers in both Tables 1 and 2) and compare it with values from other approaches. To achieve our goal of comparison, the criteria of the correlation thresholds in the *C*-threshold approach, both conditional and unconditional, is to choose such values that the generated networks have the same network density as those networks generated by the conditional *P*-threshold approach (refer to the “Generals” column in Table 1 and the “Densities” column in Table 2). In the same spirit, the overlap ratios in the last column of Table 1 stand for the percentages of overlapped edges filtered by other approaches as compared with those filtered by the conditional *P*-threshold approach (the ratios in the latter are set to be 1).

In terms of the statistical properties shown in Table 1, when changing from correlations to partial correlations in the same filtering approach, either *P*-threshold or *C*-threshold, we obtain the general trends of smaller mean values (*e.g*. from 0.227 to 0.185, in the *P*-threshold approach at the significance level of 10^−7^), smaller skewness values (*e.g*. from 1.509 to **−0.761**) and more negative edges (*e.g*. from 0.0004 to 0.0067). These trends are consistent with the observations *i-iii*) in Fig. 2. Secondly, when the significance level becomes lower, fewer edges are retained and the overlapped edges decrease. For instance, only 4.6% edges in the complete network are left in conditional *P*-threshold approach with criterion of 10^−7^ and the overlapped ratio is 0.406 using the unconditional *C*-threshold correlation approach, which means only 40.6% of edges filtered by the latter approach are significant at the level of 10^−7^. Some of the other non-overlapped edges may also be significant but not at the same level as set by the criteria. These findings confirm the observations *iv-vi*) in Fig. 2.

The implications of these findings from the perspective of the financial market are that, the positive co-movement phenomenon is a dominant feature in the whole market as negatively correlated stock pairs take extremely small portions in different types of networks. Whereas, after conditioning on the market index, the portions of negative correlated stock pairs, and also the stock pairs with smaller positive correlation values, tend to increase, so that the mean value of correlations of the whole stock network becomes smaller. Besides, these comparison results clearly show the advantage of the conditional *P*-threshold approach in filtering out all the statistically insignificant values and leave only those reliable and purely correlated stock pairs.

To show the performance of the *P*-threshold in filtering networks of different complexities, we also compare the different approaches in terms of the topological properties of the filtered networks in Table 2. The networks are constructed based on the frame of edge types defined in Table 1 and their average correlations are the same as the mean values in Table 1. In terms of the focused network properties, when changing from correlations to partial correlations in the same approach (either *P*-threshold or *C*-threshold), we can identify the general trends of smaller cluster coefficient values (*e.g*. from 0.755 to 0.417, in the *P*-threshold approach at the significance level of 10^−7^), decreased small-world tendency (*e.g*. the ratio of the average distance over the clustering coefficient, from 2.256 to 6.277) and decreased assortatitivity (*e.g*. the absolute value of coefficient, from 0.218 to 0.061). Secondly, when the significance level becomes smaller, as fewer edges are kept, the number of clusters increases and the stock network becomes less connected but more dispersed. Besides, the clustering coefficient decreases and the average distance increases, so that the small-world property is weakened. In addition, with respect to the above properties, we find that the conditional *P*-threshold approach performs similarly to the conditional *C*-threshold approach as their overlap ratios are very high (more than 90% under all criteria), but better than the unconditional *P*-threshold and unconditional *C-*threshold approaches. For instance, the ratios (measuring the small-world tendency) in the second last column in Table 2 are bigger than those in the unconditional *P*-threshold and the unconditional *C*-threshold approaches as compared to those in the conditional *P*-threshold approach.

Noticeably, the networks filtered by the conditional *P*-threshold approach are really distinguished in terms of the assortativity, as is highlighted in the last column of Table 2. Generally, when the networks become denser, the assortativity coefficients become smaller. But, compared to the unconditional *C*-threshold approach, to filter networks of the same density, the proposed conditional *P*-threshold presents less disassortative (one order of magnitude lower) structures. This indicates that the proposed conditional *P*-threshold filtering approach can reflect the reliable and well-defined hierarchical market structure with dominant assortativity features. These features can be further verified in the dynamic networks by moving the windows along the long time series.

### Results of evolutionary dynamic networks

**Figure 4:**
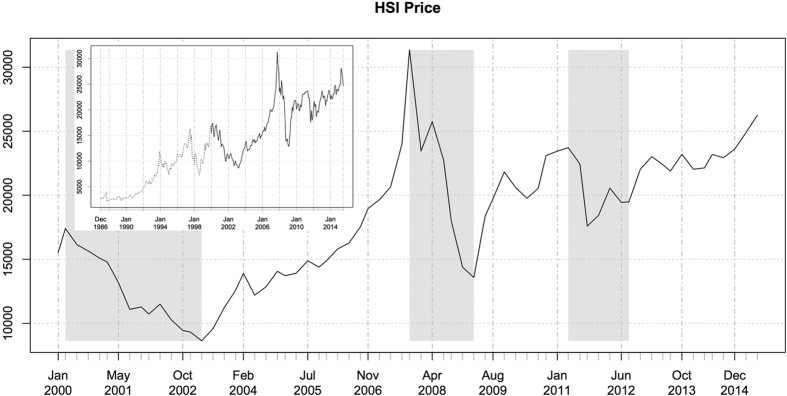
Historical records of the Hang Seng Index (HSI), where the dataset starts from January 2000 to July 2015 (the dark curve in the inset). The crises intervals on the Hong Kong stock market are highlighted with shaded bars, where the market price (HSI) fluctuates from the market peaks to the market bottoms in the special periods (from March 2000 to March 2003 as the first gray region, from October 2007 to March 2009 as the second gray region, and from April 2011 to August 2012 as the third gray region).

The time period from 2000 to 2015 covers three global financial crises, the 2001 dotcom crash (tech stock bubble bursts), the 2008 subprime crisis (global financial crisis) and the 2011 European sovereign debt crisis. In Fig. 4, the crises intervals on the Hong Kong stock market are highlighted with shaded regions, during which the market price (HSI) drops from the peaks to the bottoms (that is, from March 2000 to March 2003 in the first gray region, from October 2007 to March 2009 in the second gray region, and from April 2011 to August 2012 in the third gray region). This sufficiently long time period covers several bull runs, bear markets, and periods of “business as usual”. This demonstrates that, our network analysis represent markets of all different conditions.

To obtain evolutionary results, we adopt the strategy of moving windows to construct series of evolving networks along the observation period. In the subsequent figures, the points in the horizontal axis are the end time points of the moving windows. In each snapshot of the moving windows, we employ the proposed conditional *P*-threshold approach to form three networks (the general network *G*, the positive correlated network *PG* and the negative correlated network ***N**G*). For comparison, we also set three different significance levels for each snapshot, *P* value < 0.01, *P* value < 0.05 and *P* value < 0.1. For example, “*G (P-value < 0.01*)” stands for the general network with significantly correlated edges at ***α*** = 0.01. As most edges have positive weights, the properties of the general network and the positive network are very close to each other.

In the following subsections, we will analyze the dynamics of the Hong Kong stock network with respect to the average correlation, clustering coefficient, small-world property and degree assortativity. The definitions and detailed information of these topological characteristics can be found in the SI section. The general observational conclusion is that the dynamics of network parameters can reflect the change of the market price (here, HSI), to different extents, especially on the occasions of market crashes. And, the smaller the significance levels become, the more sensitive and reliable the network evolution properties will be. Besides, we also observe some interesting phenomena in revealing the economic changes from the expectations of the investment behaviors. Therefore, we find that some investigated network properties together can serve as a good indicator of the financial market development.

### Average correlations

**Figure 5:**
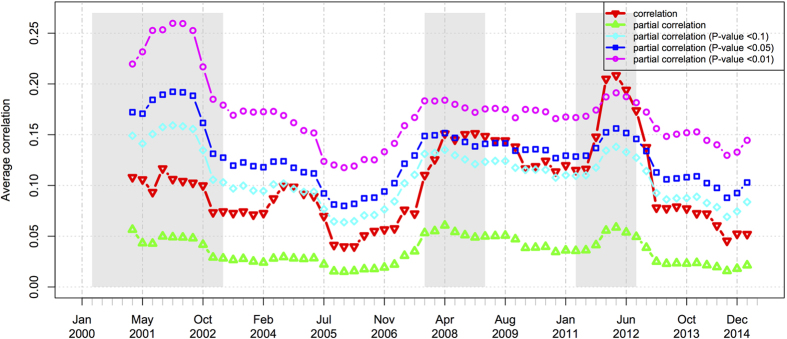
The dynamics of the average correlations of the Hong Kong stock market. The grayed regions denote the time periods of market crashes.

In Fig. 5, the line of red triangles denotes the raw unconditional correlation without removing the market index, whereas the line of green triangles denotes the partial correlation conditioning on HSI. Compared to the average raw correlation (the red line), the average partial correlation (the green line) is smaller in all windows. Besides, the average correlations show sharp increase at the beginning of the financial crisis and sharp decrease after the crisis (refer to the area when the red line intersects with the second gray region and the third gray region). This variation is evident and notable, especially during the third crisis. However, the variation becomes mild and less mitigated by conditioning on the market index (from correlation to partial correlation). Through this mitigation of the varying trend, it can be seen that the market index has a large effect on the sharp variation trend and the average partial correlation performs less acutely in reflecting the market crashes. It is still unknown whether there are other factors (such as the effect of other economic variables) that could also contribute to the fluctuation of the market structure, or it is the market crash itself that leads to the structural fluctuation so that it can be reflected in both correlation and partial correlation.

To make it more prominent, we enlarge the variation of the average partial correlation by applying the conditional *P*-threshold approach and obtain the other three indices of average partial correlation at three significant levels. In Fig. 5, the other three lines (denoted with *P* < 0.01, 0.05 or 0.1) stand for the partial correlations that are tested to be significant at the levels of 99%, 95% or 90%, respectively. It can be shown that the other three indices present the same trends as that of the average partial correlation, but in a clearer and more accurate manner, by removing the non-significant correlations. When the significance levels become stricter, the values of average partial correlations become larger yet the evolving trends in all the three significance levels are consistent in reflecting the fluctuations of the market price. Therefore, we conclude that, by comparing to the correlation and the partial correlation, the dynamics of average correlations at the three significance levels are more sensitive thereby can reflect the market crashes more clearly and more accurately.

**Figure 6:**
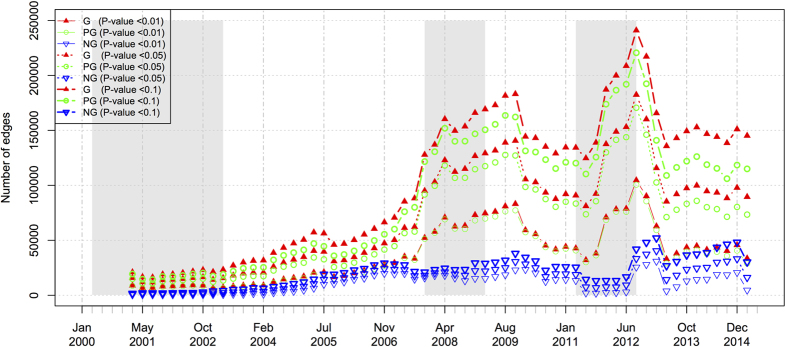
The dynamics of the number of correlated stock pairs in the Hong Kong stock market. The grayed regions denote the time periods of market crashes.

**Figure 7:**
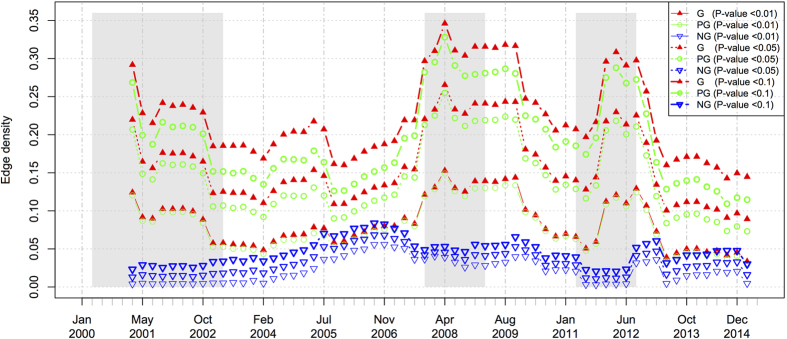
The dynamics of the density of the correlated stock pairs in the Hong Kong stock market. The grayed regions denote the time periods of market crashes.

According to Equation (15) in the SI section, the average correlation is affected by the number of edges, which may vary a lot with the moving windows due to the stocks exclusion from and inclusion into the Hong Kong Main Board. Therefore, we further delve into the dynamics of the number of edges (the number of correlated stock pairs) and the edge density of the filtered stock pairs over all possible correlated stock pairs, as shown in Figs 6 and 7, separately. In Fig. 6, the number of correlated stock pairs keeps steadily increasing for the first five years and the increase rate accelerates at the beginning of the 2008 subprime crisis. Then, the number of correlated stock pairs achieves some local peaks during the crisis, but continues to increase until three quarters after the 2008 crisis. After that, the overall correlation fades and the rising-up suddenly takes place at the beginning of the 2011 European sovereign debt crisis. Similar trends can be observed in the edge density of the positive networks in Fig. 7. But we note that the dynamics of the edge density can also reflect the 2001 dotcom crash as this metric excludes the effect of the number of stocks (the number of stocks in the Main Board is small during this crash).

### Clustering coefficients

**Figure 8:**
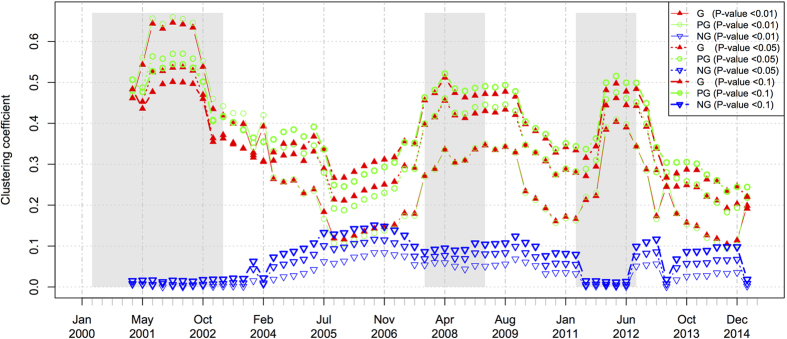
The dynamics of the clustering coefficients of the Hong Kong stock networks. The grayed regions denote the time periods of market crashes.

As for the clustering coefficient, we analyze both positive and negative networks (refer to Fig. 8). Generally, the clustering coefficient values of the positive networks and the negative networks are opposite to each other due to the complementarity of these two types of edges. That is, when the proportion of positive weighted edges increases, the proportion of negative weighted edges will decrease, and vice versa under the assumption of a fixed number of edges in the general network. Due to the opposite trends, both positive and negative networks can indicate the fluctuation of the market price in terms of the clustering coefficient, especially during the periods of the three financial crashes.

We now further elaborate on the indications of the positive networks. For the 2001 dotcom crash, the clustering coefficient of the positive networks increase at first when the market steps into crash and decreases at the end of the crash. Similarly, the clustering coefficient also serves as a good indicator for both the 2008 subprime crisis and 2011 European sovereign debt crisis. Especially, for the 2008 subprime crisis, the values of the clustering coefficient continue to be in the high state for three quarters after the end of the crisis, implying that this crisis is different from the other two crises in some sense reflected by the networking properties.

### The small-world property

**Figure 9:**
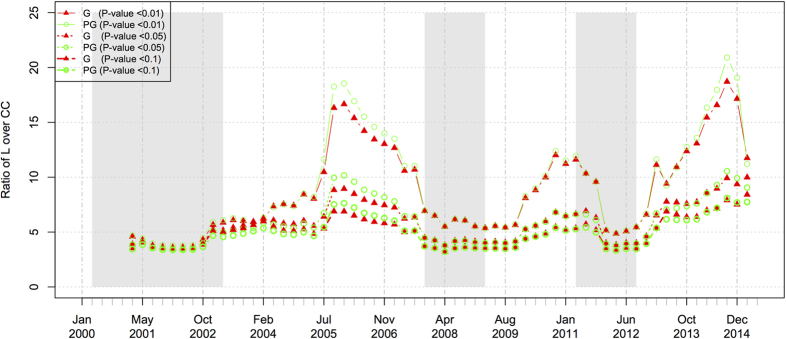
The dynamics of the small-world property of the Hong Kong stock market, measured by the ratio of the shortest path length over the clustering coefficient. The grayed regions denote the time periods of market crashes.

As for the small-world property, two network characteristics should be considered: the average (shortest) path length and the average clustering coefficient. The ratio of the path length over the clustering coefficient can measure the tendency of the small-world property. This ratio decreases when the value of the path length decreases or/and the value of clustering coefficient increases, and both cases can indicate that the small-world connectedness tendency of the network becomes more prominent. Figure 9 shows the dynamic changes of the ratios in both the general and the positive networks at the three significance levels. Note that, in this figure, we ignore the negative networks as there are fewer edges and moreover the small-world property is not prominent with respect to the other networks in consideration.

In Fig. 9, the general trends of the ratios (measuring the small-world property) at the three significance levels vary a lot. At the significance levels of 0.1 and 0.05, the ratios are generally low throughout the whole sample period. But, at the level of 0.01, the ratios become more sensitive with obvious upward trends and larger fluctuations than those at lower levels. Despite the distinct variations, one common pattern among all individual lines is that the ratio reaches its local minimums during the crash periods. This pattern manifests that, once some financial crisis happens, all ratios at different significance levels diminish to similar lower states and then stay in the lower states until the end of the crisis. Based on this observation and the last time points of the collected data, we can make a prediction that the next incoming crisis will likely happen if all the three kinds of ratios start to shrink to similar lower states again after the observed period, namely after February 2015.

### Degree assortativity

**Figure 10:**
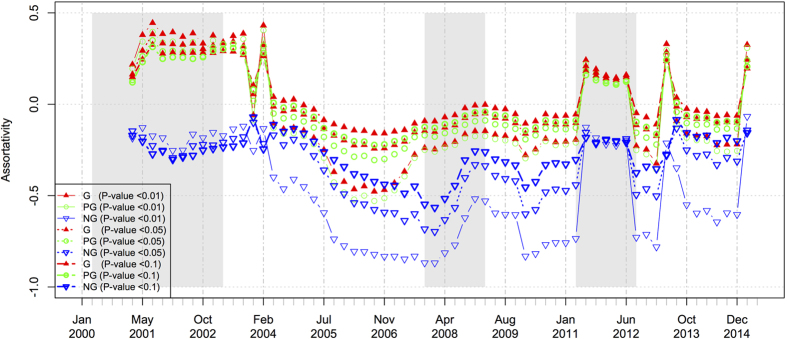
The dynamics of the degree assortativity in the Hong Kong stock market. The grayed regions denote the time periods of market crashes.

As can be observed from Fig. 10, during the normal business period, the low assortativity coefficients in the negative networks indicate that they are disassortative networks. It means that, stocks prefer connecting with other stocks that significantly differ from themselves, implying that heterogeneous stocks behave oppositely, or observing the negative co-movement behavior of heterogeneous stocks. On the other hand, during the periods of financial crises, the high assortativity coefficients in the positive networks indicate that high-degree stocks (with more correlated partners) tend to correlate with other similar high-degree stocks, implying that the homogeneous stocks increase or decrease together, or observing the positive co-movement of homogeneous stocks. This phenomenon could be related to the homogeneous expectations of investors^30^ in the stock market.

The fluctuations of degree assortativity in Fig. 10 present the repeated patterns in both the 2001 dotcom crash and the 2011 European sovereign debt crisis, which are consistent with the observations in terms of the small-world property. Specifically, all three types of networks at three different significance levels shrink to have closely similar states of the degree assortativity during financial crises. It is also interesting to note that the positive networks shrink to have the higher positive degree assortativity, whereas the negative networks shrink to have the lower negative assortativity. That is, in both the 2001 dotcom crash and the 2011 European sovereign debt crisis, the homogenous positive co-movement trend is prominent, and the heterogeneous negative co-movement trend is weakened. But, in the 2008 subprime crisis, only the weakening of the heterogeneous negative co-movement is prominent whereas the positive co-movement of homogeneous stocks does not change much. Besides, when the significance level increases, the degree assortativity becomes more sensitive, especially for the negative networks. This could be attributed to the facts that there are fewer edges in the negative network when the significance level becomes stricter.

In summary, the assortativity coefficients in the networks, especially in the negative networks, filtered by the conditional *P*-threshold approach, can manifest the financial crises in a meaningful financial sense; therefore, it can serve as a good indicator of the financial market development.

## Discussion

In this paper, we have proposed a *t*-test based *P*-threshold approach for filtering out all insignificant correlations and keeping only the significantly correlated stock pairs in order to construct a reliable stock network taking the distribution of the correlation coefficients into account. We focus on the statistically reliable connectivity between any pair of stock returns from financial time series, which reflects the underlying stock market structure accurately. On the other hand, we are interested in a filtering approach that can take into account the distribution of correlation values (measuring the connectivity), which can also be used easily. We use the partial correlation of any pair of abnormal returns for two stocks to exclude the market effect and then conduct *t*-test on the partial correlation. To illustrate the performance of our proposed approach on the understanding network complexity, we collect real data from the Hong Kong stock market and build a giant static network together with a series of evolving networks with fixed-size moving windows. By comparing our proposed approaches with the conventional *C*-threshold filtering approaches, we show that our *P*-threshold (conditional and unconditional) approaches are more effective in filtering out insignificant edges and keeping only the significant edges that can reflect the reliable “pure” correlation structure of the stocks. The 2001 dotcom crash, the 2008 subprime crisis and the 2011 European sovereign debt crisis, covered by the dataset, can be prominently indicated by our new approach in terms of the evolving network properties. Moreover, we have some interesting findings about the Hong Kong stock market, including the strengthened positive co-movement of homogeneous stocks but weakened negative co-movement of heterogeneous stocks during financial crises. In addition, we find that the 2008 subprime crisis exposes distinguished patterns from the other two crises in terms of basic network characteristics, such as clustering coefficient, small-world property and assortativity.

In this paper, we find that our proposed *P*-threshold approaches work well for the Hong Kong stock market in the period from January 2000 to July 2015. Academics and practitioners may wonder whether our proposed approaches could work well in other periods as well as other markets. Thus, future work can include the examination of our proposed approaches in other periods as well as on other stock markets and even the global finance market.

## Method

### Correlation and partial correlation

Let *p*_*i*_(*t*) and ***r***_***i***_(***t***) denote the daily closing prices and returns of stock ***i*** at time ***t** (**i*** = **1, 2, …,** ***N**, **t*** = **1, 2, …,** ***T***), respectively. The logarithmic return, ***r***_***i***_(***t***), of stock ***i*** over the passing period **Δ*****t*** is defined as:


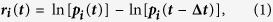


In this paper, we set **Δ*****t*** = **1**, so ***r***_***i***_(***t***) is precisely the daily return of stock ***i*** at ***t***.

The cross-correlation matrix ***C*** for all the logarithmic returns ***r***_***i***_(***t***) is computed with elements ***c***_***ij***_, which is the correlation coefficient between a stock pair ***i*** and ***j***, defined as


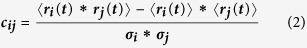


where 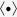 and 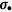 denote the mean and standard deviation of the time series, with 
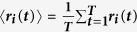
 and 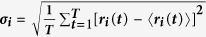.

In many situations, a large correlation value between two stocks calculated by Equation (2) does not necessarily mean that there is a strong “pure” correlation between these two stocks. Sometimes, their “pure” correlation could be insignificant or even be zero. The reason for the correlation between two stocks to be strong is because the correlation is “contaminated” by the market factor, common macroeconomic factors, and some important events, as argued by^31^,^32^. In this paper, therefore, we recommend examining the “pure” correlation between any pair of stocks by employing a partial correlation between the pair of stocks, so as to remove the market factor (represented by the market index).

According to the Capital Asset Pricing Model (CAPM), the excess return or abnormal return of a stock is obtained by removing the market return from the actual corresponding return of the stock, which will take out the systematic risk component^32^. In this paper, the systematic risk component comes from the market index, which is the Hang Seng Index (HSI), represented by ***I***. Many studies^24^,^28^ have adopted this methodology to evaluate the actual performance of a stock in the analysis of stock networks. Thereby, we also take this approach to study the following market model:


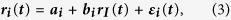


where 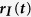 represents the corresponding daily return of the market index ***I**, **a***_***i***_ and ***b***_***i***_ are parameters in the regression, and the error term 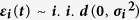.

The estimated daily return, 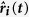, for the *i*-th stock for each *i* is obtained via


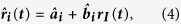


where 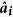 and 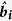 are the estimates of the parameters ***a***_***i***_ and ***b***_***i***_, respectively. Therefore, the daily stock-specific abnormal return 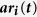 can be obtained via


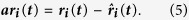


Similarly, the return of stock ***j*** is obtained via 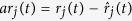.

Therefore, the partial correlation matrix 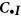 for all the abnormal returns 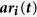 is computed with elements 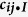, which is the partial correlation coefficient between each pair of stocks ***i*** and ***j***, defined as


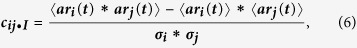


where 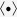 and 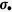 represent the mean and standard deviation of the time series, respectively, and *I* stands for Hang Seng Index, with 
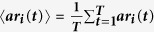
 and 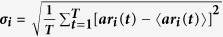.

In the rest of this section, we call Equation (6) as “partial correlation”, or “conditional correlation”, in order to differentiate it from the “unconditional Pearson correlation”, or simply “correlation”, as defined in Equation (2).

### The *C*-threshold approach

An edge in the network corresponds to an element in the (conditional) correlation matrix ***C*** (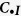), *e.g*. the edge 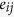 connecting nodes ***i*** and ***j*** corresponds to the element 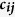 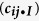. In the *C*-threshold filtering method, the criterion is set up by a threshold correlation value ***θ*****(−1** ≤ ***θ*** ≤ **1**). An edge will be chosen and added into the final network if its absolute correlation value is larger than the pre-determined threshold, *e.g*. if 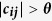 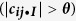. To use the (conditional) *C*-threshold filtering method, many studies (*e.g*. refs 8,29) recommend using conditional correlation matrix 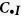 to replace the cross-correlation matrix ***C*** in the analysis.

From an econometric perspective, when estimating the correlation coefficient of two financial time series, its significance depends on the sample joint distribution and the sample size. Here, we point out two limitations of the traditional (conditional) *C*-threshold approach: (*i*) a relatively large absolute value of the (conditional) correlation coefficient may be included into the network, following the (conditional) *C*-threshold approach, but actually it is not statistically significant; and (*ii*) a relative small value of the (conditional) correlation coefficient may be discarded from the network, by the (conditional) *C*-threshold approach, but actually it is statistically significant.

To relax the limitation of the (conditional) *C*-threshold approach, in this paper we first recommend using the significance level of the correlation coefficient estimate to determine whether the corresponding edge is significant. When the correlation coefficient appears to be statistically significant, we include the edge into the network; otherwise, we exclude it from the network. As the filtering criteria are determined by the *P*-values in the hypothesis test, this method is called the *P-*threshold method. Based on different types of correlation measures, either the correlation 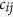 or the partial correlation 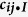, the new proposed method can be either the unconditional *P*-threshold approach or the conditional *P*-threshold approach.

### The unconditional *P*-threshold approach

We set the following hypothesis to test the null hypothesis ***H***_**0**_ that the correlation 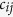 defined in Equation (2) is equal to zero versus the alternative hypothesis ***H***_**1**_ that the correlation 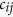 is not equal to zero:


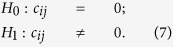


The corresponding test statistic is


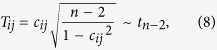


where ***n*** is the sample size and 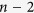 is the degree of freedom.

Given a significance level ***α***, we will reject ***H***_**0**_ if the absolute value of the test statistic in Equation (8) exceeds the critical value, denoted by 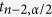, from the *t*-table evaluated at ***α***/**2**, namely, if


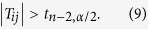


We will accept ***H***_**1**_ if the correlation between the two stocks is significant and, in this situation, we recommend the edge to be included in the stock network.

### The conditional *P*-threshold approach

We set the following hypothesis to test the null hypothesis ***H***_**0**_ that the correlation 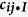 is equal to zero versus the alternative hypothesis ***H***_**1**_ that the correlation 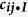 is not equal to zero:


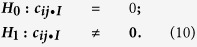


The corresponding test statistic is


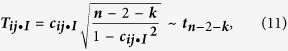


where ***n*** is the sample size, ***k*** is the number of control variables upon which the correlation is conditioned, and ***n*** − ***2*** − ***k*** is the degree of freedom. If ***H***_**0**_ is true, then as discussed above we want to condition the factor from the market index (here, HSI) thereby set ***k*** be equal to 1 in this case.

Given a significance level ***α***, we will reject ***H***_**0**_ if the absolute value of the test statistic in Equation (11) exceeds the critical value, 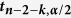, from the *t*-table evaluated at 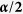, namely,


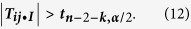


We will accept 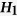 if the partial correlation between the two stocks is significant and, in this situation, we recommend the edge to be included in the stock network. The details of the network construction, and also the criteria of significance levels, will be further discussed in the next subsection.

### Network set-up

In the stock network, a node is defined as an individual stock. Traditionally, an edge connecting any two nodes is defined as the correlation coefficient of the two corresponding stock returns. Here, to construct a stock network by using the unconditional *P*-threshold approach, we recommend using the return rate of stocks and the correlation to define the edge. This can be modeled as an undirected weighted network 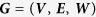, where ***V**, **E*** and ***W*** denote the sets of nodes, edges, and edge weights, respectively. By using the correlation defined in Equation (2) and the significance test in Equation (9), the matrix ***W*** = (***w***_***ij***_) with elements of the edge weight ***w***_***ij***_ is defined as follows:


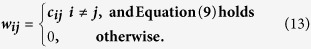


On the other hand, to construct a stock network by using the conditional *P*-threshold method, we recommend using the abnormal return rate of stocks and the partial correlation to define the edge that evaluates the “pure relationship” among the stocks after removing the market factor. This can be modeled as an undirected weighted network 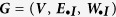, where ***V***, 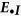 and 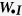 denote the sets of nodes, edges, and edge weights, respectively. By using the partial correlation defined in Equation (6) and the significance test in Equation (12), the matrix 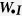 = 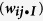 with elements of the edge weights 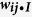 is defined as follows:


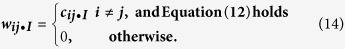


When the unconditional *P*-threshold approach is taken to capture the relation of a pair of stocks more efficiently, we further propose to divide the set of weights into two subsets, the positive set 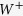
(with entry 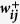, 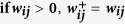; otherwise 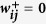), and the negative set 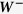 (with entry 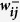, 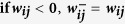; otherwise 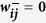), according to the signs of the estimates of the correlation coefficients.

Similarly, when the conditional *P*-threshold approach is taken to capture the “pure” relation of a pair of stocks more efficiently, we further propose to divide the set of weights into two subsets: the positive set 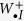 (with entry 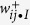, 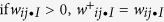; otherwise 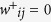), and the negative set 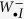 (with entry 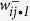, 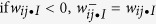; otherwise 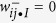), according to the signs of the estimates of the correlation coefficients.

The positive subset reflects the positive co-movement behavior whereas the negative subset characterizes the reverse behavior. The positive and negative co-movement behaviors inspire us to separate the whole stock network ***G*** into two sub-networks, a positively correlated network ***PG*** on the positive weight set 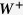 or 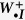 and a negatively correlated network 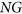 on the negative weight set 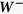 or 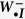. Thus, finally we obtain three types of networks: ***G**, **PG*** and ***NG***.

The filtered edges will vary with different pre-determined significance levels, and so will the topological characteristics of the networks. To verify the effectiveness of the approach, we further compare the performances of the network by setting three different significance levels: ***α*** = 0.1, 0.05, and 0.01, respectively. When the *P* value is less than 0.01, 0.05 and 0.1, respectively, we reject the null hypothesis and conclude that the partial correlation is not equal to zero.

We set a fixed window size of 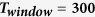 (about one trading year) with the starting point at January 3, 2000. Every time, we move the starting point forward by a fixed interval of 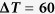 (about one trading quarter) until the whole period of 4,060 trading days is covered. We take a snapshot in each time window and construct the corresponding network there. For each rolling dynamic network, we select stocks whose trading days are not less than two thirds of the corresponding window length, which could ensure sufficient co-trading days. After processing, totally 62 snapshots are obtained and the dynamic network properties are analyzed. Within each window, there are three types of networks and each type of network is extracted at three different significance levels, thereby we construct and compare totally nine networks for each snapshot.

## Additional Information

**How to cite this article**: Xu, R. *et al*. Topological Characteristics of the Hong Kong Stock Market: A Test-based *P*-threshold Approach to Understanding Network Complexity. *Sci. Rep.* **7**, 41379; doi: 10.1038/srep41379 (2017).

**Publisher's note:** Springer Nature remains neutral with regard to jurisdictional claims in published maps and institutional affiliations.

## Supplementary Material

Supplementary Information

## Figures and Tables

**Table 1:** Statistical properties of the sixteen types of fileted edges.

		Statistic descriptions				Proportion of edges			
Edge types		Criteria	Mean values	Standard deviations	Skewnesses	Generals	Positive edges	Negative edges	Overlaps
*P-*thresholds	Correlation	10^−7^	0.227	0.071	1.509	0.373	0.9996	0.0004	0.997
	Correlation	10^−5^	0.207	0.074	1.469	0.478	0.9996	0.0004	0.998
	Correlation	10^−3^	0.184	0.077	1.390	0.628	0.9993	0.0007	0.997
	Correlation	10^−1^	0.155	0.083	1.196	0.845	0.9973	0.0027	0.989
	**Partial correlation**	**10**^**−7**^	**0**.**185**	**0**.**054**	**−0**.**761**	**0**.**046**	**0**.**9933**	**0**.**0067**	**1**
	**Partial correlation**	**10**^**−5**^	**0**.**160**	**0**.**049**	**−0**.**370**	**0**.**094**	**0**.**9932**	**0**.**0068**	**1**
	**Partial correlation**	**10**^**−3**^	**0**.**130**	**0**.**046**	**−0**.**085**	**0**.**207**	**0**.**9905**	**0**.**0095**	**1**
	**Partial correlation**	**10**^**−1**^	**0**.**090**	**0**.**050**	**0**.**154**	**0**.**529**	**0**.**9702**	**0**.**0298**	**1**
*C-*thresholds	Correlation	0.307	0.371	0.065	1.132	0.046	0.9996	0.0004	0.406
	Correlation	0.259	0.325	0.065	1.444	0.094	0.9996	0.0004	0.474
	Correlation	0.200	0.271	0.068	1.555	0.207	0.9997	0.0003	0.587
	Correlation	0.114	0.199	0.074	1.453	0.529	0.9995	0.0005	0.784
	Partial correlation	0.152	0.186	0.054	−0.815	0.046	0.9934	0.0066	0.913
	Partial correlation	0.126	0.161	0.048	−0.403	0.094	0.9933	0.0067	0.934
	Partial correlation	0.095	0.131	0.046	−0.112	0.207	0.9904	0.0096	0.957
	Partial correlation	0.048	0.090	0.050	0.139	0.529	0.9695	0.0305	0.983

As the conditional *P*-threshold approach is our main focus in this paper, we take the type of the *P*-threshold partial correlation as the basis (refer to Row Five to Row Eight, highlighted by the bold-faced numbers). The “Criteria” stands for the significance levels in *P*-threshold approach, and stands for the absolute correlation threshold values in *C-*threshold approach. The “Generals” denotes the percentage of the filtered edges as compared to the edges in the whole complete network, and the “Positive edges” plus the “Negative edges” equal to 100% of the “Generals”. The last column, “Overlaps”, stands for the percentage of overlapped edges as compared to the edges filtered by the conditional *P*-threshold approach, which is the focusing approach of this paper.

**Table 2:** Topological properties of the sixteen types of stocks networks with respect to the sixteen edge types defined in Table 1.

		Basic properties				Focused properties				
Network types		Nodes	Densities	Clusters	Ratio of nodes in largest clusters	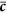	*CC*	*L*	*L*/*CC*	*r*
*P-*thresholds	Correlation ( < 10^−7^)	1279	37.3%	14	0.990	0.227	0.755	1.704	2.256	−0.218
	Correlation ( < 10^−5^)	1279	47.8%	3	0.998	0.207	0.787	1.546	1.966	−0.192
	Correlation ( < 10^−3^)	1279	62.68%	1	1.000	0.184	0.833	1.374	1.650	−0.152
	Correlation ( < 10^−1^)	1279	84.5%	1	1.000	0.155	0.909	1.155	1.270	−0.071
	**Partial correlation** (** < 10**^**−7**^)	**1279**	**4**.**60%**	**89**	**0**.**931**	**0**.**185**	**0**.**417**	**2**.**616**	**6**.**277**	**−0**.**061**
	**Partial correlation** (** < 10**^**−5**^)	**1279**	**9**.**37%**	**18**	**0**.**987**	**0**.**160**	**0**.**468**	**2**.**249**	**4**.**801**	**−0**.**056**
	**Partial correlation** (** < 10**^**−3**^)	**1279**	**20**.**7%**	**2**	**0**.**999**	**0**.**130**	**0**.**548**	**1**.**848**	**3**.**372**	**−0**.**027**
	**Partial correlation** (** < 10**^**−1**^)	**1279**	**52**.**9%**	**2**	**0**.**999**	**0**.**090**	**0**.**697**	**1**.**470**	**2**.**110**	**−0**.**016**
*C-*thresholds	Correlation (>0.307)	1279	4.60%	534	0.559	0.371	0.616	2.092	3.398	−0.331
	Correlation (>0.259)	1279	9.37%	331	0.733	0.325	0.650	2.147	3.304	−0.303
	Correlation (>0.200)	1279	20.7%	103	0.917	0.271	0.696	1.957	2.810	−0.261
	Correlation (>0.114)	1279	52.9%	2	0.999	0.199	0.799	1.482	1.855	−0.173
	Partial correlation (>0.152)	1279	4.60%	82	0.936	0.186	0.411	2.589	6.303	−0.077
	Partial correlation (>0.126)	1279	9.37%	15	0.989	0.161	0.463	2.232	4.821	−0.063
	Partial correlation (>0.095)	1279	20.7%	2	0.999	0.131	0.544	1.841	3.382	−0.028
	Partial correlation (>0.048)	1279	52.9%	2	0.999	0.090	0.694	1.471	2.119	−0.020

As the conditional *P*-threshold approach is our main focus in this paper, we take the type of the *P*-threshold partial correlation as the basis (refer to Row Five to Row Eight, highlighted by the bold-faced numbers). The focused properties are defined in Supplementary Equation (15) and Supplementary Equations (17–19). 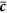 stands for the average correlation, ***CC*** is the clustering coefficient, ***L*** is the average shortest path length, ***r*** is the assortativity coefficient.
