# Supplementary material for: Topological Characteristics of the Hong Kong Stock Market: A Test-based P-threshold Approach to Understanding Network Complexity
Source: Sci Rep. 2017 Feb 1;7:41379. doi: 10.1038/srep41379 (PMC5286437; doi:10.1038/srep41379)
Supplement: Supplementary Information [file srep41379-s1.pdf]

Supplementary Information:

Topological Characteristics of the Hong Kong Stock Market: A Test-based *P*-threshold Approach to Understanding Network Complexity

Ronghua Xu

City University of Hong Kong, Hong Kong

ronghuaxu2-c@my.cityu.edu.hk

Wing-Keung Wong

Asia University, Taiwan

Lingnan University, Hong Kong

wong@asia.edu.tw

Guanrong Chen (*)

City University of Hong Kong, Hong Kong

eegchen@cityu.edu.hk

Shuo Huang

Hong Kong Baptist University, Hong Kong

soekwong@gmail.com

(*) Corresponding author: +852-3442-7922

Dataset and Supplementary Results

The dataset includes the closing prices from January 3, 2000 to July 24, 2015 (totally 4060 trading days). The prices we use in the dataset are the adjusted (for splits and dividends) prices of each stock. The starting time point is when Yahoo Finance (http://finance.yahoo. com) begins to collect the data of Main Board of Hong Kong Equity Securities. Due to the reliability and timeliness of the prices, we abstract all the 1532 stocks included in the Main Board (Excluding Depositary Receipts and Investment Companies), and collect the data from Yahoo Finance. The readers may refer to the website for the details of all the 1532 stocks in the Main Board (http://www.hkex.com.hk/eng/market/sec_tradinfo/stockcode/eisdeqty_pf.htm). The names of the 48 components of HSI from the Main Board stocks are attached in **Supplementary Table S5**.

The total observation period is 15 years, and it is separated into three shorter periods, each with the length of 5 years (from 2000 to 2005 as Window 1, from 2005 to 2010 as Window 2, and from 2010 to 2015 as Window 3). The big giant network based on the 1279 stocks in Window 3 has been shown in the main part of the paper. The results on the periods of Window 1 (containing 446 stocks) and Window 2 (containing 892 stocks) are shown below.

Results of the big static network in Window 1

The distributions of the two types of correlations are exhibited in **Supplementary Fig. 1**: the Pearson correlation without removing the market effect (denoted as

“correlation”), and the partial correlation conditioning on the market index (denoted as “partial correlation”). The distributions of the *t*-statistics and the *P* values for both correlation and partial correlation are shown in **Supplementary Fig. 2.** Furthermore, in order to understand the effect of significance levels on filtering out all insignificant edges and keep only significant reliable edges, we also compare the distributions of the two types of correlations at three significance levels, $a = 0.1$, 0.05 and 0.01, respectively. These three criteria correspond to the three vertical lines in **Supplementary Fig. 2** (b), where the correlations with the *P*-value smaller than the vertical lines are retained.

Compared to the correlations in the 15-year (January, 2000 to July, 2015) window, the correlation values in Window 1 (from January, 2000 to March, 2005) cover a smaller range of [–0.5, 1.0]. Some extremely negative correlations cannot be observed in the shorter window. But, comparing the density function of the two types of correlations in **Supplementary Fig. 1**, we confirm consistent findings of *i*-*vii*) described in the subsection of **Results of the big static network** and show that the conclusions made in Window 3 can also be applied to Window 1.

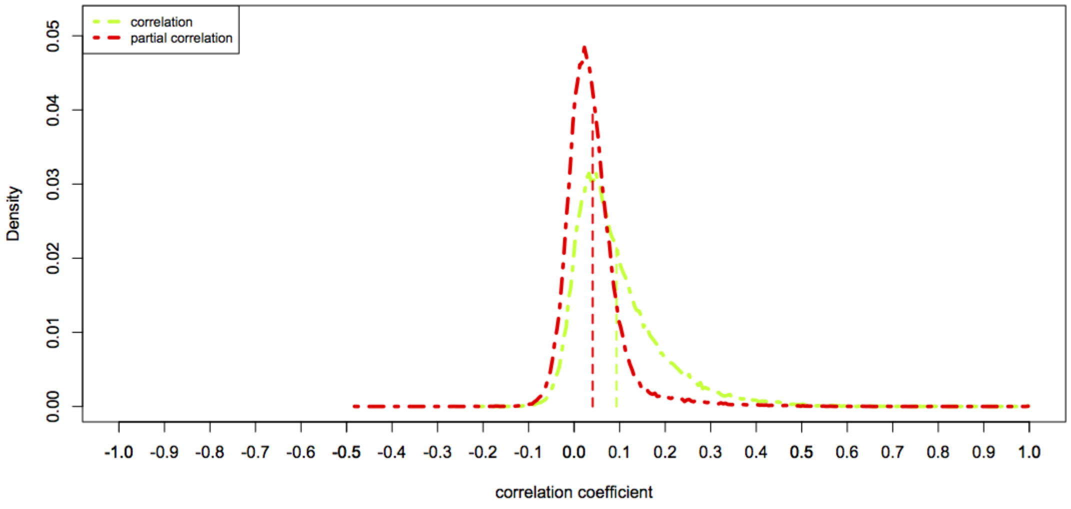
correlation
partial correlation
Density
0.00
0.01
0.02
0.03
0.04
0.05
-1.0 -0.9 -0.8 -0.7 -0.6 -0.5 -0.4 -0.3 -0.2 -0.1 0.0 0.1 0.2 0.3 0.4 0.5 0.6 0.7 0.8 0.9 1.0
correlation coefficient

(a)

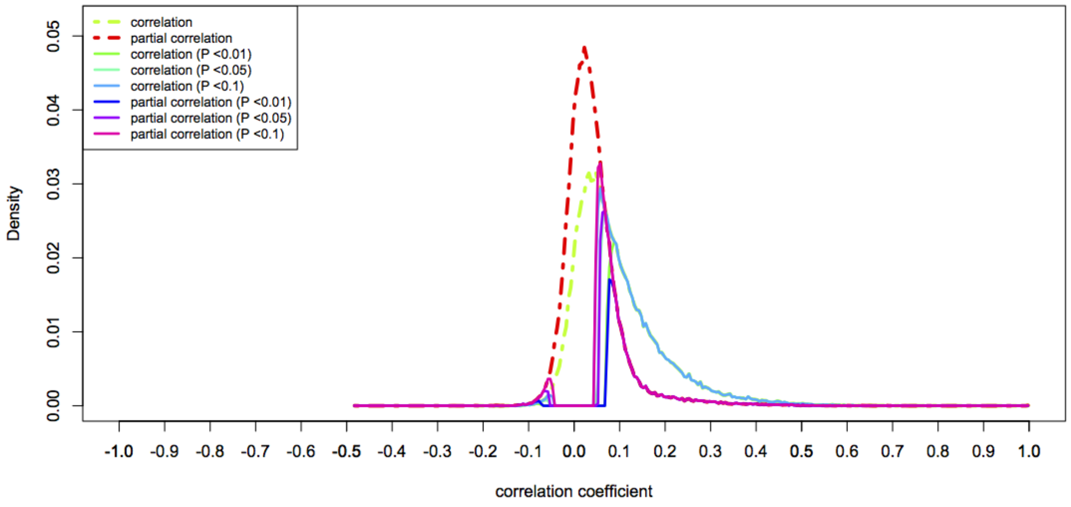
correlation
partial correlation
correlation (P <0.01)
correlation (P <0.05)
correlation (P <0.1)
partial correlation (P <0.01)
partial correlation (P <0.05)
partial correlation (P <0.1)
Density
0.00
0.01
0.02
0.03
0.04
0.05
-1.0 -0.9 -0.8 -0.7 -0.6 -0.5 -0.4 -0.3 -0.2 -0.1 0.0 0.1 0.2 0.3 0.4 0.5 0.6 0.7 0.8 0.9 1.0
correlation coefficient

(b)

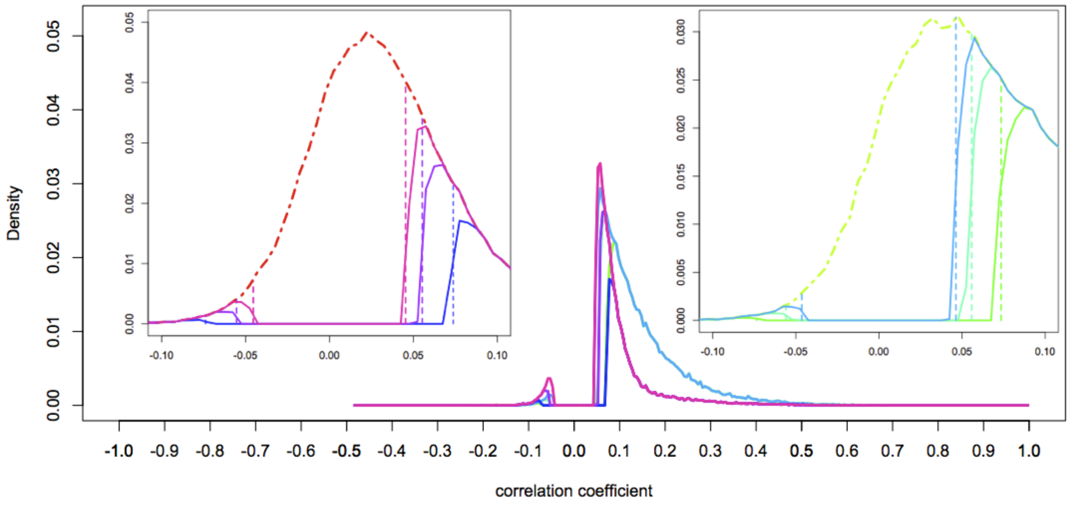

(c)

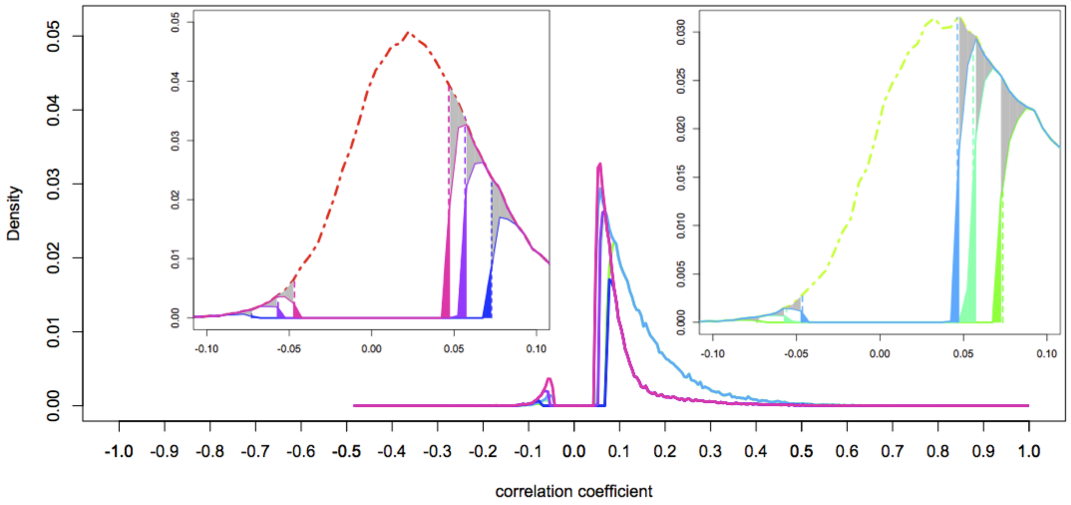

(d)

Supplementary Fig. 1. (Color online) (a) Probability density distributions of the two types of correlation values in the giant static network of 446 stocks spanning from January 03, 2000 to March 09, 2005. Here, "correlation" stands for the raw Pearson correlation coefficient (defined in Equation (2)), "partial correlation" stands for the partial correlation coefficient conditioning on HSI (defined in Equation (6)). The two vertical dotted lines, the red one and the green one, denote the mean values of "correlation" and "partial correlation", respectively. (b) Probability density distributions of the two types of correlation values in the giant static network of 446 stocks and the distributions of the filtered correlation values by the conditional and unconditional *P*-threshold approaches at three significant

levels. Here, “P <0.01”, “P <0.05”, “P <0.1” stand for the significance levels of 1%, 5% and 10%, respectively. (c) Similarly to (b) and using the same legend as (b), the distributions of the filtered correlation values by the conditional and unconditional *P*-threshold approaches at three significant levels are shown in the center of the figure. To present a clearer view, we separate and highlight the “partial correlation” (under three significance levels) coefficient and the “correlation” (under three significance levels) coefficient in the range of [−0.1, 0.1] in the left and right insets, respectively. The vertical lines in the insets denote the correlation thresholds in the *C*-threshold approach, so that the number of correlation values which are bigger than the thresholds is the same as the number of significant correlation values at the preset significant levels in the *P*-threshold approach. For example, the dark blue dotted line in the left inset denotes such correlation threshold that the number of partial correlation values bigger than this threshold is the same as the number of significant partial correlation values at the level of 1%. (d) Similarly to (c) and using the same legend as (c), the only difference is that, in the two insets, we plot out the differences between the *C*-threshold approaches and the *P*-threshold approaches by coloring the non-intersecting regions. The regions, painted with the same color as the distributions lines, denote the lower but significant correlation values that are excluded by the *C*-threshold approach but are included by the *P*-threshold approach. On the other hand, the regions, painted with the gray color, denote the regions of relative higher but non-significant correlation values that are included by the *C*-threshold approach but are excluded by the *P*-threshold approach.

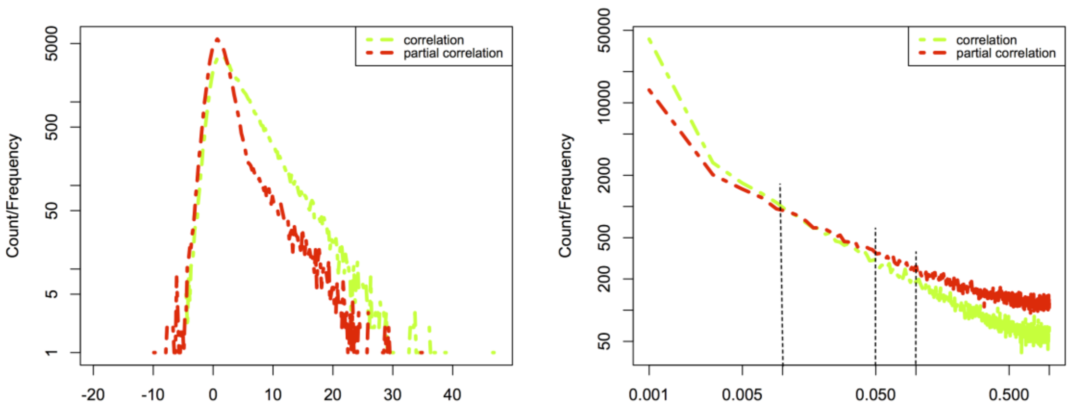

(a) (b)

Supplementary Fig. 2. (Color online) Probability density distributions of (a) *t*-statistics on weighted edges (y-axis is in log scale) and (b) the corresponding *P* values (in log-log scale). The red points denote the type of “correlation” and the green points denote the type of “partial correlation”.

We also enlarge the range of the significance levels with *P* value $<10^{-7}$, $<10^{-5}$, $<10^{-3}$ and $<10^{-1}$ in Window 1 and compare them with the *C*-threshold method. The statistical properties of the enlarged filtered edges and the network properties of the filtered stock networks are compared in **Supplementary Tables S1** and **S2**, respectively. When changing from correlations to partial correlations in the same filtering approach (either *P*-threshold or *C*-threshold), we obtain the general trends of smaller mean values, smaller skewness values, more negative edges, smaller cluster coefficient values and decreased small-world tendency, which are consistent with the observations in Window 3. Secondly, the ratios of the overlapped edges between the conditional *C*-threshold approach and the conditional *P*-threshold approach are very high (more than 95% under all criteria). Therefore, these two approaches perform very similarly in terms of both statistical and network properties. Besides, in terms of the assortativity property, the conditional correlation-based networks are assortative whereas the unconditional correlation based networks are dissasortative.

Supplementary Table S1 Statistical properties of sixteen types of fileted edges. The "Criteria" stands for the significance levels in *P*-threshold approach, and for the correlation threshold values in *C*-threshold approach. The "Generals" denotes the percentages of the edges filtered by the methods as compared to the edges in the whole complete network, and the "Positive edges" plus the "Negative edges" are equal to 100% of the "Generals". The last column, "Overlaps", stands for the percentages of overlapped edges as compared to the edges by conditional *P*-threshold approach, which is the focusing approach of this paper.

Edge types		Statistic descriptions				Proportion of edges			
		Criteria	Mean values	Standard deviations	Skewnesses	Generals	Positive edges	Negative edges	Overlaps
P-thresholds	Correlation	10^{-7}	0.239	0.088	1.724	0.213	0.9997	0.0003	0.999
	Correlation	10^{-5}	0.214	0.088	1.744	0.279	0.9996	0.0004	0.999
	Correlation	10^{-3}	0.184	0.089	1.729	0.391	0.9987	0.0013	0.997
	Correlation	10^{-1}	0.138	0.092	1.597	0.637	0.9870	0.0130	0.975
	Partial correlation	10^{-7}	0.249	0.103	1.039	0.044	0.9938	0.0062	1
	Partial correlation	10^{-5}	0.216	0.101	1.295	0.062	0.9940	0.0060	1
	Partial correlation	10^{-3}	0.163	0.095	1.608	0.117	0.9876	0.0124	1
	Partial correlation	10^{-1}	0.091	0.079	1.994	0.370	0.9449	0.0551	1
C-thresholds	Correlation	0.292	0.379	0.081	1.890	0.044	1.0000	0.0000	0.663
	Correlation	0.260	0.348	0.083	1.799	0.062	1.0000	0.0000	0.646
	Correlation	0.204	0.292	0.085	1.743	0.117	0.9999	0.0001	0.657
	Correlation	0.099	0.189	0.089	1.719	0.370	0.9985	0.0015	0.750
	Partial correlation	0.153	0.250	0.103	1.041	0.044	0.9938	0.0062	0.962
	Partial correlation	0.127	0.217	0.102	1.263	0.062	0.9933	0.0067	0.954
	Partial correlation	0.095	0.164	0.095	1.569	0.117	0.9864	0.0136	0.954
	Partial correlation	0.048	0.091	0.079	1.972	0.370	0.9434	0.0566	0.969

Supplementary Table S2 Topological properties of the sixteen types of stocks networks with respect to the sixteen edge types defined in **Supplementary Table S1**. The focused properties are defined in Supplementary Equation (15-19). $\bar{c}$ stands for the average correlation, CC is the clustering coefficient, L is the average shortest path length, and r is the assortativity coefficient.

Network types		Basic properties				Focused properties				
		Nodes	Densities	Clusters	Ratio of nodes in largest clusters	$\bar{c}$	CC	L	L/CC	r
P-thresholds	Correlation ($<10^{-7}$)	446	21.3%	78	0.818	0.239	0.744	1.932	2.596	-0.253
	Correlation ($<10^{-5}$)	446	27.9%	41	0.901	0.214	0.764	1.842	2.410	-0.208
	Correlation ($<10^{-3}$)	446	39.1%	1	1.000	0.184	0.785	1.725	2.198	-0.122
	Correlation ($<10^{-1}$)	446	63.7%	1	1.000	0.138	0.815	1.363	1.673	0.016
	Partial correlation ($<10^{-7}$)	446	4.39%	170	0.581	0.249	0.755	2.782	3.687	**0.325**
	Partial correlation ($<10^{-5}$)	446	6.21%	96	0.780	0.216	0.680	2.688	3.955	**0.229**
	Partial correlation ($<10^{-3}$)	446	11.7%	6	0.989	0.163	0.577	2.302	3.986	**0.154**
	Partial correlation ($<10^{-1}$)	446	37.0%	2	0.998	0.091	0.577	1.629	2.822	**0.128**
C-thresholds	Correlation (>0.292)	446	4.39%	252	0.415	0.379	0.669	1.880	2.811	-0.140
	Correlation (>0.260)	446	6.21%	226	0.484	0.348	0.675	1.865	2.763	-0.207
	Correlation (>0.204)	446	11.7%	165	0.621	0.292	0.710	1.837	2.587	-0.255
	Correlation (>0.099)	446	37.0%	2	0.998	0.189	0.776	1.761	2.268	-0.128
	Partial correlation (>0.153)	446	4.39%	165	0.612	0.250	0.760	3.127	4.115	0.355
	Partial correlation (>0.127)	446	6.21%	92	1.784	0.217	0.687	2.723	3.964	0.262
	Partial correlation (>0.095)	446	11.7%	4	0.993	0.164	0.580	2.286	3.938	0.189
	Partial correlation (>0.048)	446	37.0%	2	0.998	0.091	0.571	1.630	2.854	0.138

Results of the big static network in Window 2

Similart to the results in Window1, the distributions of the two types of correlation values in Window 2 (from March, 2005 to May, 2010) are exhibited in **Supplementary Fig. 3** and the distributions of the *t*-statistics and the *P* values for both correlation and partial correlation are shown in **Supplementary Fig. 4.** These results are consistent with those in both Window 1 and Window 3.

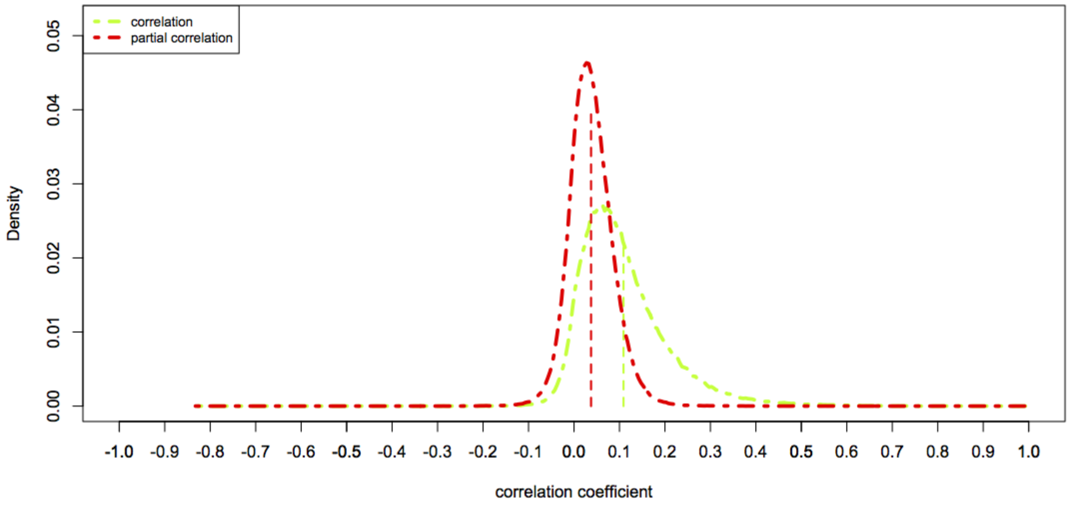

(a)

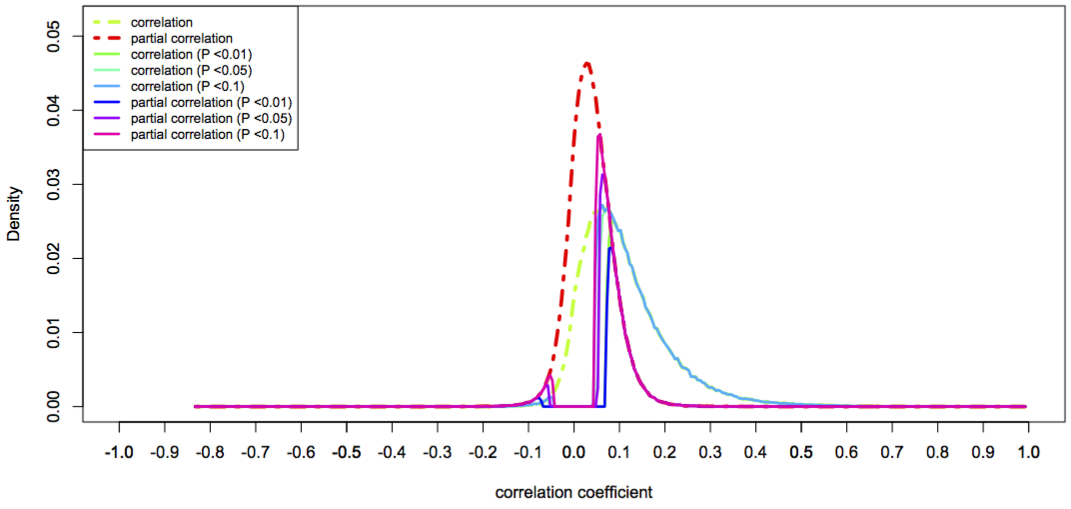

(b)

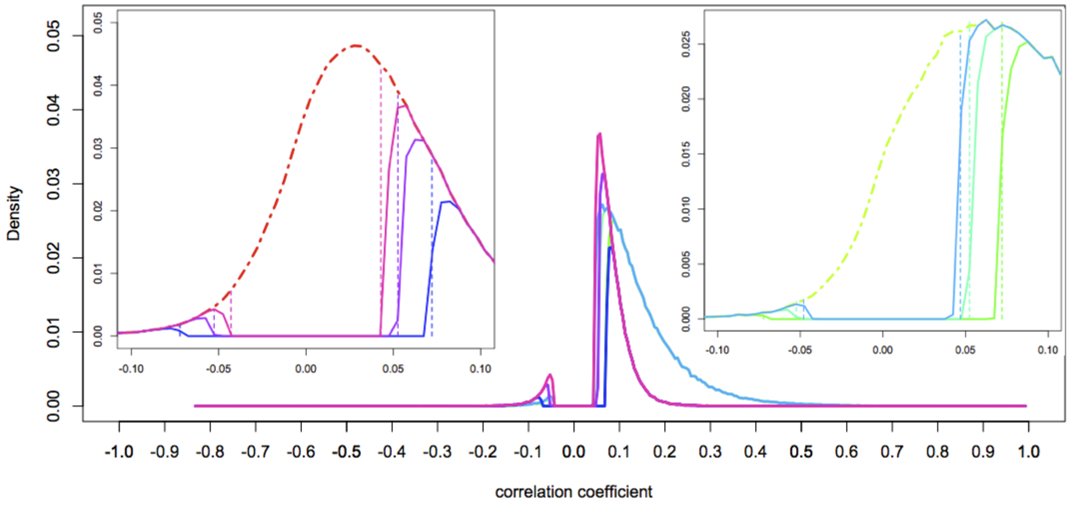

(c)

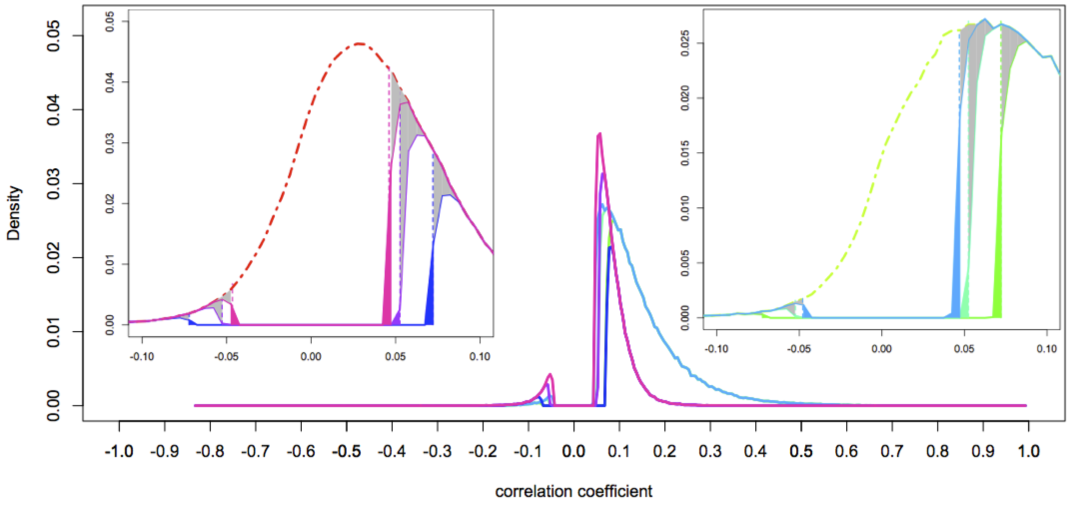

(d)

Supplementary Fig. 3. (Color online) (a) Probability density distributions of the two types of correlation values in the giant static network of 892 stocks spanning from March 10, 2005 to May 17, 2010. Here, “correlation” stands for the raw Pearson correlation coefficient (defined in Equation (2)), “partial correlation” stands for the partial correlation coefficient conditioning on HSI (defined in Equation (6)). The two vertical dotted lines, the red one and the green one, denote the mean values of “correlation” and “partial correlation”, respectively. (b) Probability density distributions of the two types of correlation values in the giant static network of 892 stocks and the distributions of the filtered

correlation values by the conditional and unconditional *P*-threshold approaches at three significant levels. Here, “P <0.01”, “P <0.05”, “P <0.1” stand for the significance levels of 1%, 5% and 10%, respectively. (c) Similarly to (b) and using the same legend as (b), the distributions of the filtered correlation values by the conditional and unconditional *P*-threshold approaches at three significant levels are shown in the center of the figure. To present a clearer view, we separate and highlight the “partial correlation” (under three significance levels) coefficient and the “correlation” (under three significance levels) coefficient in the range of [−0.1, 0.1] in the left and right insets, respectively. The vertical lines in the insets denote the correlation thresholds in the *C*-threshold approach, so that the number of correlation values which are bigger than the thresholds is the same as the number of significant correlation values at the preset significant levels in the *P*-threshold approach. For example, the dark blue dotted line in the left inset denotes such correlation threshold that the number of partial correlation values bigger than this threshold is the same as the number of significant partial correlation values at the level of 1%. (d) Similarly to (c) and using the same legend as (c), the only difference is that, in the two insets, we plot out the differences between the *C*-threshold approaches and the *P*-threshold approaches by coloring the non-intersecting regions. The regions, painted with the same color as the distributions lines, denote the lower but significant correlation values that are excluded by the *C*-threshold approach but are included by the *P*-threshold approach. On the other hand, the regions, painted with the gray color, denote the regions of relative higher but non-significant correlation values that are included by the *C*-threshold approach but are excluded by the *P*-threshold approach.

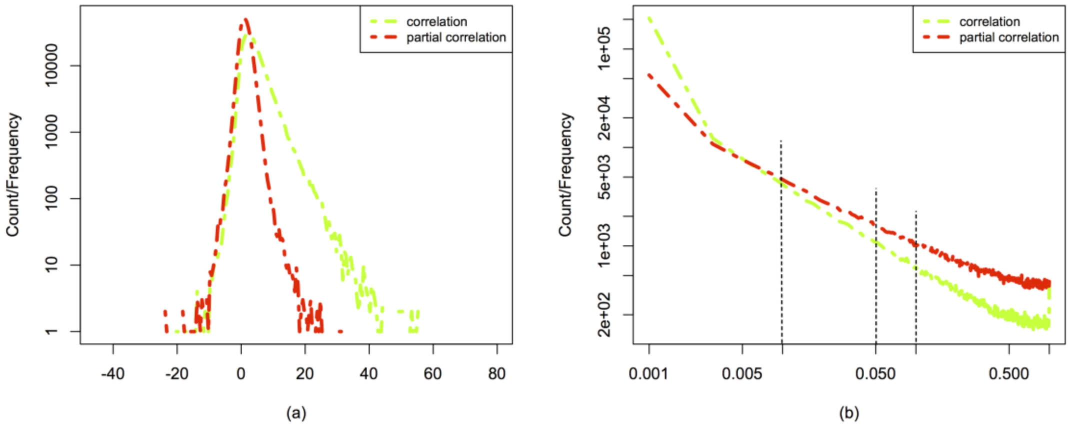

Supplementary Fig. 4. (Color online) Probability density distributions of (a) *t*-statistics on weighted edges (y-axis is in log scale) and (b) the corresponding *P* values (in log-log scale). The red points denote the type of "correlation" and the green points denote the type of "partial correlation".

The statistical properties of the enlarged filtered edges in Window 2 and the network properties of the filtered stock networks are compared in **Supplementary Tables S3** and **S4**, respectively. When changing from correlations to partial correlations in the same approach (either *P*-threshold or *C*-threshold), we obtain the general trends of smaller mean values, smaller skewness values, more negative edges, smaller cluster coefficient values and decreased small-world tendency, which are consistent with the observations in both Window 1 and Window 3. Secondly, the ratios of the overlapped edges between the conditional *C*-threshold and the conditional *P*-threshold approaches are very high. Therefore, these two approaches perform very similarly. Besides, in terms of the assortativity property, the conditional correlation-based networks are assortative whereas the unconditional correlation based networks are dissasortative.

Supplementary Table S3 Statistical properties of sixteen types of fileted edges. The "Criteria" stands for the significance levels in *P*-threshold approach, and for the correlation threshold values in *C*-threshold method. The "Generals" denotes the percentages of the edges filtered by the methods as compared to the edges in the whole complete network, and the "Positive edges" plus the "Negative edges" are equal to 100% of the "Generals". The last column, "Overlaps", stands for the percentages of overlapped edges as compared to the edges by conditional *P*-threshold approach, which is the focusing approach of this paper.

Edge types		Statistic descriptions				Proportion of edges			
		Criteria	Mean values	Standard deviations	Skewnesses	Generals	Positive edges	Negative edges	Overlaps
P-thresholds	Correlation	10^{-7}	0.235	0.088	1.495	0.262	0.9986	0.0014	0.978
	Correlation	10^{-5}	0.210	0.088	1.546	0.348	0.9979	0.0021	0.983
	Correlation	10^{-3}	0.181	0.089	1.579	0.488	0.9963	0.0037	0.981
	Correlation	10^{-1}	0.142	0.092	1.510	0.739	0.9880	0.0120	0.966
	Partial correlation	10^{-7}	0.169	0.103	-2.069	0.017	0.9499	0.0501	1
	Partial correlation	10^{-5}	0.145	0.080	-2.094	0.040	0.9598	0.0402	1
	Partial correlation	10^{-3}	0.116	0.062	-1.815	0.114	0.9628	0.0372	1
	Partial correlation	10^{-1}	0.073	0.052	-0.771	0.415	0.9343	0.0657	1
C-thresholds	Correlation	0.388	0.471	0.089	-1.954	0.017	0.9981	0.0019	0.298
	Correlation	0.312	0.398	0.089	-0.037	0.040	0.9987	0.0013	0.346
	Correlation	0.221	0.307	0.088	1.128	0.114	0.9989	0.0011	0.461
	Correlation	0.108	0.195	0.088	1.580	0.415	0.9973	0.0027	0.705
	Partial correlation	0.152	0.171	0.102	-2.133	0.017	0.9523	0.0477	0.883
	Partial correlation	0.126	0.146	0.080	-2.141	0.040	0.9604	0.0396	0.903
	Partial correlation	0.094	0.116	0.061	-1.847	0.114	0.9632	0.0368	0.935
	Partial correlation	0.047	0.074	0.052	-0.779	0.415	0.9341	0.0659	0.977

Supplementary Table S4 Topological properties of the sixteen types of stocks networks with respect to the sixteen edge types defined in **Supplementary Table S3**. The focused properties are defined in Supplementary Equation (15-19). $\bar{c}$ stands for the average correlation, CC is the clustering coefficient, L is the average shortest path length, and r is the assortativity coefficient.

Network types		Basic properties				Focused properties				
		Nodes	Densities	Clusters	Ratio of nodes in largest clusters	$\bar{c}$	CC	L	L/CC	r
P-thresholds	Correlation ($<10^{-7}$)	892	26.2%	30	0.965	0.235	0.732	1.886	2.576	-0.242
	Correlation ($<10^{-5}$)	892	34.8%	6	0.994	0.210	0.747	1.734	2.321	-0.196
	Correlation ($<10^{-3}$)	892	48.8%	2	0.999	0.181	0.778	1.523	1.957	-0.127
	Correlation ($<10^{-1}$)	892	73.9%	1	1.000	0.142	0.852	1.261	1.481	-0.037
	Partial correlation ($<10^{-7}$)	892	1.71%	114	0.867	0.169	0.330	3.249	9.858	**0.263**
	Partial correlation ($<10^{-5}$)	892	3.99%	23	0.975	0.145	0.333	2.609	7.835	**0.188**
	Partial correlation ($<10^{-3}$)	892	11.4%	2	0.999	0.116	0.386	2.000	5.182	**0.142**
	Partial correlation ($<10^{-1}$)	892	41.5%	2	0.999	0.073	0.561	1.584	2.822	**0.079**
C-thresholds	Correlation (>0.388)	892	1.71%	621	0.288	0.471	0.680	2.312	3.401	-0.343
	Correlation (>0.312)	892	3.99%	500	0.426	0.398	0.672	2.152	3.209	-0.393
	Correlation (>0.221)	892	11.4%	226	0.738	0.307	0.697	2.174	3.118	-0.348
	Correlation (>0.108)	892	41.5%	3	0.998	0.195	0.762	1.620	2.128	-0.161
	Partial correlation (>0.152)	892	1.71%	114	0.864	0.171	0.325	3.181	9.781	0.204
	Partial correlation (>0.126)	892	3.99%	24	0.974	0.146	0.333	2.589	7.776	0.144
	Partial correlation (>0.094)	892	11.4%	2	0.999	0.116	0.387	1.996	5.159	0.133
	Partial correlation (>0.047)	892	41.5%	2	0.999	0.074	0.561	1.585	2.827	0.076

Topological characteristics

The average correlation is defined to be the mean of all the non-diagonal elements of the correlation matrix, as follows:

$$\bar{c} = \frac{1}{|E|} \sum_{(i,j)\in E} c_{ij\bullet I} \qquad (15)$$

where $|E|$ is the number of edges in a network. The average correlation measures the overall level of correlations among connected pairs of stocks in the network.

In a network, a node i has k_i neighbors and thus the degree of the node is k_i. There are at most $k_i(k_i - 1)/2$ edges among all the neighbors. Let e_i denote the number of connected pairs existing among these neighbors. The clustering coefficient CC_i of node i is defined to be the ratio of its actual number of edges over the maximum number of possible edges:

$$CC_i = \frac{2e_i}{k_i(k_i - 1)} . \qquad (16)$$

Subsequently, the network clustering coefficient, CC, is the average of the clustering coefficients of all nodes:

$$CC = \frac{1}{|N|} \sum_{i=1}^{|N|} CC_i . \qquad (17)$$

The clustering coefficient of a stock network reveals the clustering property of stocks in the sense of price fluctuation correlation, and shows the tendency that the prices of a bunch of stocks increase or decrease together.

For the entire network, it is possible to represent the geodesic distances by a distance matrix, in which the entry d_{ij} represents the length of the shortest path between nodes i and j. The average shortest path length is defined as follows:

$$L = \frac{2}{|N|(|N|-1)} \sum_{i<j} d_{ij} \ , \qquad (18)$$

where the sum accounts for all distances with $i \neq j$ and disregards pairs that are not in the same connected component.

In addition, the assortativity of a network is used to detect the correlations of node degrees, thereby inferring the hierarchical structure of the network, which is defined as follows:

$$r = \frac{|E|^{-1} \sum_{(i,j)\in E} k_i k_j - \left[|E|^{-1} \sum_{(i,j)\in E} \frac{1}{2}(k_i + k_j) \right]^2}{|E|^{-1} \sum_{(i,j)\in E} \frac{1}{2}(k_i^2 + k_j^2) - \left[|E|^{-1} \sum_{(i,j)\in E} \frac{1}{2}(k_i + k_j) \right]^2} \ , \qquad (19)$$

where k_i and k_j are the degrees of the end nodes of edge $(i,j) \in E$, and $|E|$ is the number of edges in the network.

Clearly, r ranges from –1 to 1. If $r > 0$, then the network is assortative, implying that the nodes with large degrees tend to connect to nodes also with large degrees on average. This implies that a “big” stock, which co-moves with a large number of other stocks, tends to connect to other “big” stocks. That is, homogeneous stocks tend to connect together. If $r < 0$, then it is disassortative, meaning that the nodes with large degrees tend to connect to nodes with small degrees on average. Similarly, its implication in a stock network is that a “big” stock tends to connect to “small” stocks that co-move with a small number of stocks. This also means that heterogeneous

stocks tend to connect together. If $r = 0$, then the nodes degrees in the network are uncorrelated on average.

Supplementary Tables

Supplementary Table S5. The stock symbols and names of the 48 components of HSI from the Main Board.

Symbol	Name	Symbol	Name	Symbol	Name
0001.HK	CHK HOLDINGS	0135.HK	KUNLUN ENERGY	0939.HK	CCB
0002.HK	CLP HOLDINGS	0144.HK	CHINA MER HOLD	0941.HK	CHINA MOBILE
0003.HK	HK & CHINA GAS	0151.HK	WANT WANT CHINA	0992.HK	LENOVO GROUP
0004.HK	WHARF HOLDINGS	0267.HK	CITIC	1044.HK	HENGAN INT’L
0005.HK	HSBC HOLDINGS	0291.HK	CHINA RES BEER	1088.HK	CHINA SHENHUA
0006.HK	POWER ASSETS	0293.HK	CATHY PAC AIR	1109.HK	CHINA RES LAND
0011.HK	HANG SENG BANK	0322.HK	TINGYI	1299.HK	AIA
0012.HK	HENDERSON LAND	0386.HK	SINOPEC CORP	1398.HK	ICBC
0016.HK	SHK PPT	0388.HK	HKEX	1880.HK	BELLE INT’L
0017.HK	NEW WORLD DEV	0494.HK	LI & FUNG	1928.HK	SANDS CHINA LTD
0019.HK	SWIRE PACIFIC A	0688.HK	CHINA OVERSEAS	2318.HK	PING AN
0023.HK	BANK OF E ASIA	0700.HK	TENCENT	2319.HK	MENGNIU DIARY
0027.HK	GALAXY ENT	0762.HK	CHINA UNICOM	2388.HK	BOC HONG KONG
0066.HK	MTR CORPORATION	0836.HK	CHINA RES POWER	2628.HK	CHINA LIFE
0083.HK	SINO LAND	0857.HK	PETROCHINA	3328.HK	BANKCOMM
0101.HK	HANG LONG PPT	0883.HK	CNOOC	3988.HK	BANK OF CHINA
